# Key comorbidities in the development of post-COVID conditions: diabetes mellitus, hypertension, and obesity

**DOI:** 10.1590/1980-220X-REEUSP-2025-0473en

**Published:** 2026-08-21

**Authors:** Karina Marques Prediger, Ana Cristina Ribeiro La Scaléa, Sílvia Carla da Silva André Uehara

**Affiliations:** 1 Universidade Federal de São Carlos, Departamento de Enfermagem, São Carlos, SP, Brazil.

**Keywords:** Post-Acute COVID-19 Syndrome, Prevalence, Diabetes Mellitus, Hypertension, Obesity

## Abstract

**Objective:**

To analyze and compare the symptoms of post-COVID condition (PCC) in patients with and without chronic comorbidities, such as diabetes, hypertension, and obesity.

**Method:**

This is a cross-sectional and analytical study, considering a sample of 350 patients with PCC treated in Primary Health Care services. Data were collected from medical records and analyzed using a Poisson regression model to estimate the prevalence ratio.

**Results:**

The prevalence of PCC symptoms was reduced by 50% for headache and by 64% for sore throat in non-diabetic individuals. People with hypertension showed twice the prevalence of muscle weakness and/or loss of strength compared to those with normal blood pressure. In addition, diarrhea and upper limbs tingling/throbbing were more prevalent in obese individuals.

**Conclusion:**

The study presented a bidirectional relationship between PCC and pre-existing chronic conditions, providing a detailed understanding of how specific comorbidities influence PCC symptoms.

## INTRODUCTION

Since the beginning of the COVID-19 pandemic, it has been identified that people with metabolic diseases have an increased risk of developing a severe form of the disease and of death compared to people without comorbidities. Furthermore, it was observed that some of the comorbid individuals infected with SARS-CoV-2 presented a more favorable profile for the development of PCC^(1,2,3)^.

PCC is defined by the World Health Organization (WHO) as a disease characterized by symptoms that develop in individuals with a history of COVID-19, beginning three months after the acute illness and which cannot be explained by an alternative diagnosis. Symptoms of PCC can begin after the acute phase of the initial illness, fluctuate or relapse over time, and the most common are fatigue, shortness of breath, and headache^(4)^.

In the literature, it is possible to find other synonymous terms for PCC, such as long COVID, “post-acute COVID-19”, “post-COVID syndrome”, “long-term effects of COVID”, “post-acute COVID-19 syndrome”, among other names. In Brazil, the Ministry of Health (MS) adopted the term “post-COVID condition” to standardize technical documents and guide healthcare professionals on the subject^(5)^. According to Technical Note No. 57/2023 from the MS, PCC is characterized by the presence of signs and/or symptoms that develop four weeks or more after initial SARS-CoV-2 infection and cannot be explained by an alternative diagnosis. Furthermore, the post-COVID condition has the potential to improve, worsen, or relapse over time, and may evolve into serious events months or years after the acute infection^(6)^.

It is estimated that 20 to 30% and 50 to 89% of non-hospitalized and hospitalized COVID-19 patients, respectively, are susceptible to developing PCC symptoms^(7)^. It is known that female sex, advanced age, pre-existing medical conditions, lack of COVID-19 vaccination, number of symptoms during the acute phase, viral load, severe course during the acute phase of the disease, and use of invasive mechanical ventilation are factors that indicate a greater propensity to present PCC symptoms^(1,2)^.

It is suggested that long-term symptoms following recovery from the acute phase of COVID-19 are associated with the virus ability to trigger a massive inflammatory response. Frequently, PCC symptoms are also related to the severity of the acute phase of the disease, but they can also occur after a mild manifestation of COVID-19, as well as affect patients of all ages^(8)^. Furthermore, the PCC symptoms can even worsen pre-existing symptoms of comorbidities previously present in individuals^(1)^.

Due to the characteristics of PCC symptoms, which are common to other diseases, the diagnosis of this condition is often overlooked and complex. Currently, the greatest challenge in treating patients with PCC is the fact that this disease cannot be considered a single clinical entity, requiring integrated multidisciplinary treatment due to the diversity of symptoms^(2)^. Therefore, understanding the course of this disease is critical for recovery and for improving people’s quality of life.

In addition, the etiology of PCC has to be fully understood in light of the diverse symptoms and phenotypes. To date, the literature has shown that PCC is mainly associated with female sex, advanced age, socioeconomic vulnerability, COVID-19 vaccination status, and pre-existing clinical conditions such as diabetes, hypertension, and obesity^(2,3,8,9)^. However, studies comparing the manifestation of PCC symptoms between individuals with and without chronic comorbidities are still scarce, hindering the comprehension of the influence of these conditions on symptoms persistence. Therefore, this study aims to analyze and compare the symptoms of post-COVID condition in patients with and without chronic comorbidities, such as diabetes, hypertension, and obesity.

## METHOD

### Design of Study

This is a cross-sectional and analytical study, guided by the recommendations of the Strengthening the Reporting of Observational Studies in Epidemiology (STROBE).

### Local

This study was conducted in the municipality of São Carlos, located in the state of São Paulo. Data were obtained from the Health Surveillance services and the Department of Outpatient Management and Care, the sector responsible for Primary Health Care (PHC) services.

### Population and Selection Criteria

A sample calculation was performed considering all COVID-19 cases diagnosed in the population over 18 years of age in the municipality, related to the period from 02/26/2020 to 02/26/2024, through Rapid Test (RT) or Reverse Transcription Polymerase Chain Reaction (RT-PCR). In the city of São Carlos-SP, 62,977 positive cases of COVID-19 were reported in individuals over 18 years of age during the analysis period of this study. The choice of prevalence was based on data from the literature^(10)^, which indicate that approximately 50% of COVID-19 cases presented with PCC symptoms. The study included adult patients over 18 years of age who tested positive for COVID-19 and returned to PHC services with symptoms of PCC, as defined by the WHO, which describes symptoms as recurrent or fluctuating for a minimum period of two months after infection with the acute disease^(4)^. Patients whose medical records were inaccessible, who did not reside in areas covered by primary health care, or who had died were excluded from the study. The study variables were sex, age, race/color, comorbidities, vaccination schedule, and reported symptoms.

### Sample Size Calculation

For the sample size calculation, a significance level of 5% (α = 0.05), a tolerable absolute error of 5 percentage points (d = 0.05), and a finite population estimated at N = 31,489 patients with PCC over 18 years of age in the municipality of interest were assumed. The expected prevalence used was p = 0.356, corresponding to the frequency of the symptom fatigue, one of the most frequently reported manifestations in previous studies on PCC. Thus, the sample size adopted in this study was n = 350. The calculation was performed using the following equation:


$$n=\frac{z_{\frac{\left(1-\alpha \right)}{2}}^{2}Np(1-p)}{d^{2}(N-1)+z_{\frac{\left(1-\alpha \right)}{2}}^{2}p(1-p)}$$

Where p is the expected prevalence, d is the tolerable absolute error, N is the population size, and z is the value of the standard normal distribution corresponding to the desired confidence level.

### Data Collection

For data collection, spreadsheets were created in Microsoft Excel, containing the variables of interest. Initially, different tabs were created to collect data from the various databases used in this research, obtained from *SisCOVID*, the COVID-19 notification system of the municipality of São Carlos-SP. In the next tab, data regarding vaccination status, obtained from *VaciVida*, a system developed by the State of São Paulo, were entered. Finally, in the last tab, the data regarding PCC symptoms obtained from the Citizen’s Electronic Medical Record (PEC) were entered. Measures were adopted to minimize bias, including training for the data collectors (two undergraduates, two master’s students, and one doctoral student in Health Sciences), use of predefined inclusion and exclusion criteria, and standardization of data collection through a structured and digitized spreadsheet. Furthermore, the following stand out: choosing the appropriate significance level (α = 0.05), adjusting for multiple comparisons, applying appropriate statistical methods, and using a robust sample size.

Thus, first, confirmed cases of COVID-19 were identified in *SisCOVID*; subsequently, the PEC was consulted to determine if the patient infected with SARS-CoV-2 had returned to PHC reporting PCC symptoms, according to the WHO definition^(4)^. Through *SisCOVID*, it was also possible to obtain information about consultations with users who complained of respiratory symptoms after the acute phase of the disease; while through *PEC*, PCC symptoms reported by users, which affected different organ systems of the body, were verified. Furthermore, vaccination data were obtained from the *VaciVida* website, in conjunction with the municipal epidemiological surveillance agency.

### Data Analysis and Treatment

Initially, data were described using absolute and percentage frequencies (qualitative variables) and measures of central tendency and dispersion, such as mean, standard deviation, minimum, median, and maximum (quantitative variables). To estimate the crude prevalence ratio of PCC symptoms in relation to the variables of interest, the Poisson regression model with robust variance was used^(11)^. In analyses where the frequency of symptoms was zero, Fisher’s exact test was performed. The use of *Missing Data* was indicated in the table footnotes to ensure transparency of the data presented, maintaining the number of missing cases as observed, without imputation. All analyses were performed using the software SAS 9.4^(12)^, adopting a significance level of 5%.

### Ethical Aspects

In accordance with Resolution No. 510/2016, the project was approved by the Research Ethics Committee of the Universidade Federal de São Carlos (CAAE: 67128023.4.0000.5504; CAAE: 74911623.5.0000.5504).

## RESULTS

To obtain the sample of 350 post-COVID cases, 3,778 reported and confirmed cases of COVID-19 were analyzed, corresponding to 9.26% of acute disease cases in the adult population of the municipality of São Carlos-SP. The results show that 67.1% (235) were female, 61.6% (215) were white, 48.2% (169) had comorbidities, with 30.3% (106) having Systemic Arterial Hypertension (SAH), 15.1% (53) having Diabetes Mellitus (DM), and 16% (56) having obesity (16%), and the mean age was 46.9 years (Table 1).

**Table 1:** Demographic and clinical characterization of the study sample – São Carlos, SP, Brazil, 2024.

Characteristics	Frequency (n = 350)	Percentage (%)
**Sex**		
Female	235	67.1
Male	115	32.9
**Age**		
Mean	46.9 [15.8]	–
Median (Minimum and Maximum)	47.0 [14.0 and 88.0]	–
**Age range**		
< 60 years	270	77.1%
60 years and older	80	22.9%
**Race/Color**		
Yellow	1	0.3
White	215	61.6
Brown	105	30.1
Black	28	8.0
Missing	1	–
**Comorbidities**		
Hemoglobinopathies	1	0.3
Liver cirrhosis	0	0
Cardiovascular diseases	17	4.9
Neurological diseases	4	1.1
Diabetes Mellitus (Type 1 and Type 2)	53	15.1
Chronic Kidney Disease	4	1.1
Systemic Arterial Hypertension	106	30.3
Immunosuppressed	0	0
Obesity	56	16
Severe chronic lung diseases	21	6.0
Others	51	14.6

The prevalence of PCC symptoms, considering the absence or presence of comorbidities, was reduced by 58% for runny nose (95% CI 0.23;0.78), by 31% for headache (95% CI 0.49;0.98), and by 46% for sore throat (95% CI 0.34;0.85) in people without comorbidities (Table 2).

**Table 2:** Comparison of the presence of post-COVID condition symptoms, considering the presence of comorbidities in the study sample – São Carlos, SP, Brazil, 2024.

Description of symptoms of the post-COVID condition	Non-Comorbid (n = 181)	Comorbid (n = 169)	Prevalence ratio [CI 95%]*	p-value^†^
Changes in menstruation^‡^	6 (3.6%)	2 (1.2%)	0.34 (0.07; 1.68)	0.19
Anxiety^‡^	11 (6.6%)	10 (6.2%)	0.94 (0.41; 2.15)	0.88
Tiredness	32 (17.1%)	25 (14.8%)	0.84 (0.52; 1.35)	0.47
Runny nose	33 (18.2%)	13 (7.7%)	0.42 (0.23; 0.78)	<0.01
Diarrhea	1 (0.6%)	3 (1.8%)	3.21 (0.34; 30.59)	0.31
Difficulty concentrating^‡^	3 (1.8%)	2 (1.2%)	0.69 (0.12; 4.06)	0.68
Abdominal pain^‡^	12 (7.2%)	9 (5.6%)	0.77 (0.33; 1.79)	0.55
Joint pain^‡^	4 (2.4%)	3 (1.9%)	0.77 (0.18; 3.4)	0.73
Headache	59 (32.6%)	38 (22.5%)	0.69 (0.49; 0.98)	0.04
Sore throat	44 (24.3%)	22 (13.0%)	0.54 (0.34; 0.85)	<0.01
Pain in lower limbs^‡^	14 (8.4%)	25 (15.4%)	1.84 (0.99; 3.41)	0.05
Pain in upper limbs^‡^	6 (3.6%)	9 (5.6%)	1.55 (0.56; 4.25)	0.40
Muscle pain	28 (15.5%)	23 (13.6%)	0.88 (0.53; 1.47)	0.62
Back/lower back pain^‡^	19 (11.4%)	11 (6.8%)	0.6 (0.29; 1.21)	0.15
Earache^‡^	0 (0%)	1 (0.6%)	–	0.49^§^
Chest pain^‡^	8 (4.8%)	9 (5.6%)	1.16 (0.46; 2.93)	0.75
Chest pain^‡^	8 (4.8%)	5 (3.1%)	0.64 (0.22; 1.93)	0.43
Shortness of breath	22 (12.2%)	34 (20.1%)	1.66 (1.01; 2.71)	0.05
Lower limbs tingling/throbbing^‡^	7 (4.2%)	9 (5.6%)	1.33 (0.51; 3.47)	0.57
Upper limbs tingling/throbbing^‡^	6 (3.6%)	9 (5.6%)	1.55 (0.56; 4.25)	0.40
Muscle weakness/loss of strength^‡^	12 (7.2%)	18 (11.1%)	1.55 (0.77; 3.11)	0.22
Insomnia^‡^	9 (5.4%)	10 (6.2%)	1.15 (0.48; 2.75)	0.76
Fever	19 (10.5%)	15 (8.9%)	0.85 (0.44; 1.61)	0.61
Nausea	6 (3.3%)	2 (1.2%)	0.36 (0.07; 1.74)	0.20
Odynophagia^‡^	2 (1.2%)	0 (0%)	–	0.50^§^
Memory loss/confusion^‡^	5 (3.0%)	10 (6.2%)	2.06 (0.72; 5.9)	0.18
Loss of smell	5 (2.8%)	2 (1.2%)	0.43 (0.08; 2.18)	0.31
Loss of taste	3 (1.7%)	1 (0.6%)	0.36 (0.04; 3.4)	0.37
Hair loss^‡^	19 (11.4%)	11 (6.8%)	0.6 (0.29; 1.21)	0.15
Dental complaint of post-COVID tooth loss^‡^	2 (1.2%)	1 (0.6%)	0.52 (0.05; 5.63)	0.59
Sadness/depression^‡^	5 (3.0%)	10 (6.2%)	2.06 (0.72; 5.9)	0.18
Dizziness^‡^	7 (4.2%)	8 (4.9%)	1.18 (0.44; 3.17)	0.75
Cough	44 (24.3%)	42 (24.9%)	1.02 (0.71; 1.48)	0.91
Vomiting	9 (5.0%)	3 (1.8%)	0.36 (0.1; 1.3)	0.12

*IC = Confidence Interval;

^†^p-value = Significance level;

^‡^Missing 20;

^§^Value related to Fisher’s exact test.

Among the most frequent comorbidities in the study population, the relationship between the presence of PCC symptoms and SAH, DM, and obesity was analyzed, with greater emphasis on the first one. Thus, with regard to DM, it is noteworthy that anxiety was only reported in the sample that did not present this comorbidity. The prevalence of PCC symptoms was reduced by 50% for headache (95% CI 0.26;0.98) and by 64% for sore throat (95% CI 0.14;0.95) in non-diabetic individuals (Table 3).

**Table 3:** Comparison of the presence of post-COVID condition symptoms considering the diagnosis of diabetes mellitus in the study population – São Carlos, SP, Brazil, 2024.

Description of symptoms of the post-COVID condition	Non-diabetics [A] (n = 297)	Diabetics [B] (n = 53)	Prevalence ratio [B/A] [CI 95%]*	p-value^†^
Changes in menstruation^‡^	8 (2.9%)	0 (0%)	–	0.36^§^
Anxiety^‡^	21 (7.6%)	0 (0%)	–	0.03^§^
Tiredness	49 (16.5%)	8 (15.1%)	0.91 (0.46; 1.82)	0.80
Runny nose	44 (14.9%)	2 (3.8%)	0.25 (0.06; 1.02)	0.05
Diarrhea	2 (0.7%)	2 (3.8%)	5.6 (0.81; 38.92)	0.08
Difficulty concentrating^‡^	5 (1.8%)	0 (0%)	–	0.99^§^
Abdominal pain^‡^	17 (6.2%)	4 (7.5%)	1.23 (0.43; 3.5)	0.70
Joint pain^‡^	5 (1.8%)	2 (3.8%)	2.08 (0.42; 10.46)	0.37
Headache	89 (30.0%)	8 (15.1%)	0.5 (0.26; 0.98)	0.04
Sore throat	62 (20.9%)	4 (7.5%)	0.36 (0.14; 0.95)	0.04
Pain in lower limbs^‡^	33 (12.0%)	6 (11.3%)	0.95 (0.42; 2.15)	0.90
Pain in upper limbs^‡^	10 (3.6%)	5 (9.4%)	2.6 (0.93; 7.31)	0.07
Muscle pain	46 (15.5%)	5 (9.4%)	0.61 (0.25; 1.46)	0.27
Back/lower back pain^‡^	25 (9.1%)	5 (9.4%)	1.04 (0.42; 2.6)	0.93
Earache^‡^	0 (0%)	1 (1.9%)	–	0.16^§^
Chest pain^‡^	15 (5.4%)	2 (3.8%)	0.69 (0.16; 2.95)	0.62
Chest pain^‡^	12 (4.3%)	1 (1.9%)	0.43 (0.06; 3.27)	0.42
Shortness of breath	44 (14.8%)	12 (22.6%)	1.53 (0.87; 2.7)	0.14
Lower limbs tingling/throbbing^‡^	11 (4.0%)	5 (9.4%)	2.37 (0.86; 6.53)	0.10
Upper limbs tingling/throbbing^‡^	7 (2.5%)	4 (7.5%)	2.98 (0.9; 9.81)	0.07
Muscle weakness/loss of strength^‡^	22 (8.0%)	8 (15.1%)	1.89 (0.89; 4.02)	0.10
Insomnia^‡^	17 (6.2%)	2 (3.8%)	0.61 (0.15; 2.57)	0.50
Fever	32 (10.8%)	2 (3.8%)	0.35 (0.09; 1.42)	0.14
Nausea	8 (2.7%)	0 (0%)	–	0.61^§^
Odynophagia^‡^	2 (0.7%)	0 (0%)	–	0.99^§^
Memory loss/confusion^‡^	12 (4.3%)	3 (5.7%)	1.3 (0.38; 4.46)	0.67
Loss of smell	7 (2.4%)	0 (0%)	–	0.60^§^
Loss of taste	4 (1.3%)	0 (0%)	–	0.99^§^
Hair loss^‡^	25 (9.1%)	5 (9.4%)	1.04 (0.42; 2.6)	0.93
Dental complaint of post-COVID tooth loss^‡^	3 (1.1%)	0 (0%)	–	0.99^§^
Sadness/depression^‡^	13 (4.7%)	2 (3.8%)	0.8 (0.19; 3.45)	0.77
Dizziness^‡^	12 (4.3%)	3 (5.7%)	1.3 (0.38; 4.46)	0.67
Cough	75 (25.3%)	11 (20.8%)	0.82 (0.47; 1.44)	0.49
Vomiting	11 (3.7%)	1 (1.9%)	0.51 (0.07; 3.86)	0.51

*IC = Confidence Interval;

^†^p-value = Significance level;

^‡^Missing 20;

^§^Value related to Fisher’s exact test.

The results show that hypertensive individuals had twice the prevalence of manifesting weakness and/or loss of muscle strength compared to normotensive individuals (95% CI 1.13; 4.38); furthermore, hypertensive individuals did not report the presence of nausea, odynophagia, and vomiting (Table 4).

**Table 4:** Comparison of the presence of post-COVID condition symptoms considering the diagnosis of SAH in the study population – São Carlos, SP, Brazil, 2024.

Description of symptoms of the post-COVID condition	Normotensive [A] (n = 244)	Hypertensive [B] (n = 106)	Prevalence ratio [B/A] [CI 95%]*	p-value^†^
Changes in menstruation^‡^	7 (3.1%)	1 (1.0%)	0.32 (0.04; 2.55)	0.28
Anxiety^‡^	15 (6.6%)	6 (5.9%)	0.89 (0.36; 2.23)	0.80
Tiredness	40 (16.4%)	17 (16.0%)	0.98 (0.58; 1.64)	0.93
Runny nose	34 (14.0%)	12 (11.3%)	0.81 (0.44; 1.5)	0.50
Diarrhea	3 (1.2%)	1 (0.9%)	0.77 (0.08; 7.29)	0.82
Difficulty concentrating^‡^	4 (1.8%)	1 (1.0%)	0.56 (0.06; 4.92)	0.60
Abdominal pain^‡^	13 (5.7%)	8 (7.8%)	1.37 (0.59; 3.2)	0.47
Joint pain^‡^	5 (2.2%)	2 (2.0%)	0.89 (0.18; 4.51)	0.89
Headache	71 (29.1%)	26 (24.5%)	0.84 (0.57; 1.24)	0.39
Sore throat	51 (20.9%)	15 (14.2%)	0.68 (0.1; 1.15)	0.15
Pain in lower limbs^‡^	22 (9.7%)	17 (16.7%)	1.72 (0.96; 3.1)	0.07
Pain in upper limbs^‡^	10 (4.4%)	5 (4.9%)	1.11 (0.39; 3.17)	0.84
Muscle pain	36 (14.8%)	15 (14.2%)	0.96 (0.55; 1.67)	0.88
Back/lower back pain^‡^	23 (10.1%)	7 (6.9%)	0.68 (0.3; 1.53)	0.35
Earache^‡^	0 (0%)	1 (1.0%)	–	0.31^§^
Chest pain^‡^	13 (5.7%)	4 (3.9%)	0.68 (0.23; 2.05)	0.50
Chest pain^‡^	12 (5.3%)	1 (1.0%)	0.19 (0.02; 1.41)	0.10
Shortness of breath	35 (14.3%)	21 (19.8%)	1.38 (0.85; 2.26)	0.20
Lower limbs tingling/throbbing^‡^	11 (4.8%)	5 (4.9%)	1.01 (0.36; 2.84)	0.98
Upper limbs tingling/throbbing^‡^	8 (3.5%)	3 (2.9%)	0.83 (0.23; 3.08)	0.79
Muscle weakness/loss of strength^‡^	15 (6.6%)	15 (14.7%)	2.23 (1.13; 4.38)	0.02
Insomnia^‡^	11 (4.8%)	8 (7.8%)	1.62 (0.67; 3.9)	0.28
Fever	21 (8.6%)	13 (12.3%)	1.43 (0.74; 2.74)	0.29
Nausea	8 (3.3%)	0 (0%)	–	0.11^§^
Odynophagia^‡^	2 (0.9%)	0 (0%)	–	0.99^§^
Memory loss/confusion^‡^	8 (3.5%)	7 (6.9%)	1.95 (0.73; 5.23)	0.19
Loss of smell	5 (2.0%)	2 (1.9%)	0.92 (0.18; 4.67)	0.92
Loss of taste	3 (1.2%)	1 (0.9%)	0.77 (0.08; 7.29)	0.82
Hair loss^‡^	25 (11.0%)	5 (4.9%)	0.45 (0.18; 1.13)	0.09
Dental complaint of post-COVID tooth loss^‡^	2 (0.9%)	1 (1.0%)	1.11 (0.1; 12.13)	0.93
Sadness/depression^‡^	8 (3.5%)	7 (6.9%)	1.95 (0.73; 5.23)	0.19
Dizziness^‡^	10 (4.4%)	5 (4.9%)	0.45 (0.18; 1.13)	0.09
Cough	55 (22.5%)	31 (29.2%)	1.3 (0.89; 1.89)	0.18
Vomiting	12 (4.9%)	0 (0%)	–	0.02^§^

*IC = Confidence Interval;

^†^p-value = Significance level;

^‡^Missing 20;

^§^Value related to Fisher’s exact test.

People with obesity did not report the presence of runny nose, earache, insomnia, loss of smell and taste, dental complaints, or vomiting. In addition, diarrhea (95% CI 1.67;148.7) and upper limbs tingling/throbbing (95% CI 1.89;18.9) were more prevalent in obese individuals. On the other hand, the prevalence of PCC symptoms in obese individuals was reduced by 59% for headache (95% CI 0.2;0.83) and by 84% for sore throat (95% CI 0.04;0.65) compared to eutrophic individuals (Table 5).

**Table 5:** Comparison of the presence of post-COVID condition symptoms, considering the obesity diagnosis of the study sample – São Carlos, SP, Brazil, 2024.

Description of symptoms of the post-COVID condition	Eutrophic [A] (n = 294)	Obese [B] (n = 56)	Prevalence ratio [B/A] [CI 95%]*	p-value^†^
Changes in menstruation^‡^	7 (2.6%)	1 (1.8%)	0.71 (0.09; 5.67)	0.75
Anxiety^‡^	15 (5.5%)	6 (109%)	1.99 (0.81; 4.91)	0.13
Tiredness	51 (17.3)	6 (10.7%)	0.62 (0.28; 1.37)	0.24
Runny nose	46 (15.7%)	0 (0%)	–	<0.01^§^
Diarrhea	1 (0.3%)	3 (5.4%)	15.75 (1.67; 148.7)	0.02
Difficulty concentrating^‡^	4 (1.5%)	1 (1.8%)	1.25 (0.14; 0.93)	0.84
Abdominal pain^‡^	20 (7.3%)	1 (1.8%)	0.25 (0.03; 1.82)	0.17
Joint pain^‡^	6 (2.2%)	1 (1.8%)	0.83 (0.1; 6.76)	0.86
Headache	90 (30.6%)	7 (1.5%)	0.41 (0.2; 0.83)	0.01
Sore throat	64 (21.8%)	2 (3.6%)	0.16 (0.04; 0.65)	0.01
Pain in lower limbs^‡^	29 (10.6%)	10 (18.2%)	1.72 (0.89; 3.32)	0.11
Pain in upper limbs^‡^	11 (4.0%)	4 (7.3%)	1.81 (0.6; 5.48)	0.29
Muscle pain	47 (16.0%)	4 (7.1%)	0.45 (0.17; 1.19)	0.11
Back/lower back pain^‡^	26 (9.5%)	4 (7.3%)	0.77 (0.28; 2.11)	0.61
Earache^‡^	1 (0.4%)	0 (0%)	–	0.99^§^
Chest pain^‡^	16 (5.8%)	1 (1.8%)	0.31 (0.04; 2.3)	0.25
Chest pain^‡^	12 (4.4%)	1 (1.8%)	0.42 (0.06; 3.13)	0.39
Shortness of breath	51 (17.3%)	5 (8.9%)	0.51 (0.22; 1.23)	0.14
Lower limbs tingling/throbbing^‡^	12 (4.4%)	4 (7.3%)	1.66 (0.56; 4.96)	0.36
Upper limbs tingling/throbbing^‡^	5 (1.8%)	6 (10.9%)	5.98 (1.89; 18.9)	<0.01
Muscle weakness/loss of strength^‡^	25 (9.1%)	5 (9.1%)	1 (0.4; 2.49)	0.99
Insomnia^‡^	19 (6.9%)	0 (0%)	–	0.05^§^
Fever	33 (11.2%)	1 (1.8%)	0.16 (0.02; 1.14)	0.07
Nausea	6 (2.0%)	2 (3.6%)	1.75 (0.36; 8.45)	0.49
Odynophagia^‡^	2 (0.7%)	0 (0%)	–	0.99^§^
Memory loss/confusion^‡^	11 (4.0%)	4 (7.3%)	1.81 (0.6; 5.48)	0.29
Loss of smell	7 (2.4%)	0 (0%)	–	0.60^§^
Loss of taste	4 (1.4%)	0 (0%)	–	0.99^§^
Hair loss^‡^	25 (9.1%)	5 (9.1%)	1 (0.4; 2.49)	0.99
Dental complaint of post-COVID tooth loss^‡^	3 (1.1%)	0 (0%)	–	0.99^§^
Sadness/depression^‡^	12 (4.4%)	3 (5.5%)	1.25 (0.36; 4.27)	0.73
Dizziness^‡^	13 (4.7%)	2 (3.6%)	0.77 (0.18; 3.3)	0.72
Cough	78 (26.5%)	8 (14.3%)	0.54 (0.28; 1.05)	0.07
Vomiting	12 (4.1%)	0 (0%)	–	0.23^§^

*IC = Confidence Interval;

^†^p-value = Significance level;

^‡^Missing 20;

^§^Value related to Fisher’s exact test.

## DISCUSSION

This analysis showed that individuals without DM had a reduced prevalence of symptoms such as headache and sore throat, while hypertensive individuals showed a greater propensity for muscle weakness. Obese individuals had a higher prevalence of developing diarrhea and upper limbs tingling, but a lower prevalence of headache and sore throat. These results highlight the influence of comorbidities on PCC symptoms, suggesting that the presence of DM, SAH, and obesity can modify the symptomatic profile according to each condition. Similar to the results of this study, the literature also highlights that the most frequent comorbidities in individuals with PCC are DM, SAH, and obesity^(13)^.

In this context, seeking to clarify the relationships established between the presence of comorbidities, such as DM, SAH and obesity, and the occurrence of PCC symptoms, an association with the chronic inflammatory process after SARS-CoV-2 infection is suggested. In addition to the increased prevalence of people with DM experiencing COVID-19 worsening, this group is more frequently affected by PCC, making them more susceptible to long-term consequences than people without DM. It is known that patients with DM present with chronic inflammation due to impaired insulin signaling, resulting in a decrease in anti-inflammatory cytokines and increased expression of pro-inflammatory cytokines, such as tumor necrosis factor alpha (TNF-α), interleukin 6 (IL-6), and interleukin beta (IL-1β). Thus, with the activity of cytokines, insulin signaling is inhibited; consequently, there is an increase in insulin resistance, promoting respiratory failure and cardiopulmonary collapse^(3)^.

It is well known that the COVID-19 pandemic influenced the control of DM, as well as the progression of pre-diabetes and an increase in the number of cases of the disease due to the use of corticosteroids, resulting from high-dose therapy that led to severe hyperglycemia^(1,14,15)^. Agonists of medications used for glycemic control, such as Pioglitazone, increase the secretion of the ACE2 gene, and there is evidence that this medication inhibits the secretion of pro-inflammatory cytokines and increases anti-inflammatory cytokines, in addition to attenuating lung injury and reducing the pulmonary fibrotic reaction. Furthermore, people with diabetes and those on Metformin are more likely to experience reduced COVID-19 infections^(3,16,17)^ and, consequently, it may influence the absence of development of PCC symptoms.

A study in the United Kingdom showed that people with DM (type 1 or 2) were associated with a higher probability of a recorded diagnosis of PCC. When analyzing the prevalence of PCC among men with DM2 and their matched controls, it was lower in matched controls, 0.46%, compared to 0.54% in men with DM2 (p = 0.008). Moreover, the aforementioned study indicated that the prevalence of PCC was 0.54% in men with DM2 and 0.53% in women with DM2, with no statistically significant difference between genders. One hypothesis to explain the similarity in prevalence between men and women with DM and PCC symptoms could be related to the regular monitoring of their chronic condition, which makes them potentially more likely to report symptoms, including those related to PCC, thus increasing the probability of detecting this condition^(18)^.

In this study, people without DM showed decreased prevalences for the development of headache and sore throat as symptoms of PCC. The pathophysiology of pain in PCC is multifactorial and involves several mechanisms. Persistent pain in patients with PCC can be explained by factors such as systemic and central nervous system inflammation, which sensitize pain receptors, as well as disturbances in cerebral perfusion and direct neuronal damage caused by the virus. Changes in neurotransmitters, such as serotonin and dopamine, and psychological factors, such as stress and anxiety, also worsen the pain. These mechanisms, whether isolated or combined, contribute to persistent pain^(19)^.

In the United States, an analysis of people with DM who had COVID-19 indicated that inadequate glycemic control and elevated glycated hemoglobin (HbA1c ≥ 8%) increase the risk of the onset of new, recurrent symptoms of PCC, especially respiratory symptoms and mental confusion, between 30 and 180 days after COVID-19 infection, suggesting persistent sequelae associated with acute infections. However, the mechanisms for these findings are hypothetical, pointing to a reduction in oxidative stress and inflammation; consequently, treatment of the chronic disease would contribute to reducing the risks of developing PCC^(20)^.

Regarding the analysis of hypertension and PCC, in this study, hypertensive individuals showed a higher prevalence of development of symptoms of weakness and/or loss of muscle strength compared to normotensive individuals. A study conducted in Spain showed that hypertension was not associated with any specific symptoms of PCC; however, a higher proportion of hypertensive patients reported ≥ 3 symptoms of PCC compared to normotensive patients^(21)^. Musculoskeletal manifestations associated with PCC include myalgia, joint pain, and muscle weakness. Patients who used corticosteroids during the acute phase of the disease and people admitted to intensive care units (ICUs) may develop muscle weakness up to a year after infection. Muscle weakness has also been observed in individuals with mild initial COVID-19, although it is unclear whether this is solely a result of muscle deconditioning or disuse^(22)^.

The mechanisms underlying muscle weakness are not yet fully understood, but some possible explanations include direct damage to motor neurons and adjacent muscles caused by the virus or by the immune response during acute COVID-19 infection. Additionally, the virus can damage mitochondria and interfere with the electron transport chain, which is responsible for energy production in muscles. This process contributes to increased fatigue, weakness, and reduced exercise tolerance^(23)^.

In the context of hospitalization due to COVID-19, prolonged ICU stay was associated with substantial muscle mass loss, with an average reduction of 18.5% in rectus femoris muscle volume by the 7th day of hospitalization. It is also noted that deep sedation, essential for the implementation of Invasive Mechanical Ventilation (IMV), compromises cardiopulmonary function and induces a state of total immobility, which negatively affects skeletal muscles. When sedation alone is not sufficient to ensure mechanical ventilation, neuromuscular blocking agents are used to synchronize the patient with the ventilator, but these agents interrupt the transmission of impulses in muscle cells. Persistent neuromuscular blockade can result in prolonged paralysis, myopathy, and generalized muscle weakness^(24)^. Thus, the muscular sequelae observed in PCC result from persistent muscle dysfunction that begins in the acute phase of the disease, mainly due to decreased muscle protein synthesis, aggravated by a hyperinflammatory state, hypoxemia, infection of muscle cells, and dysregulation of the Renin-Angiotensin System (RAS).

Regarding obese individuals, this study showed a higher prevalence of diarrhea and tingling and/or throbbing sensations in the upper limbs. It was found that patients with a baseline Body Mass Index (BMI) in the overweight or obese range had an increased risk of presenting PCC symptoms, with those with a BMI greater than 30 kg/m^2^ having a 10% increased risk of reporting PCC symptoms compared to patients with a BMI between 18.5 and 25 kg/m^2(25)^.

Similarly, obesity is characterized by the development of a chronic inflammatory state, considered a key component in insulin resistance. Consequently, the expansion of adipose tissue occurs due to caloric overload and increased infiltration of immune cells, promoting a subsequent pro-inflammatory response through increased TNF-α, IL1β, or IL-6. Furthermore, activation of the Toll-Like receptor (TLR) due to infection by pathogens such as SARS-CoV-2 induces inflammation, being expressed in skeletal muscle and adipose tissue^(26)^.

Since SARS-CoV-2 is an enveloped virus, meaning it is surrounded by a lipid bilayer, its lipid metabolism plays an important role in the viral life cycle. Accordingly, the virus uses the lipid envelope to promote invasion of the host, aiming at lipid synthesis and modification of the signal of host cells, culminating in the “cytokine storm” due to the excessive activation of immune cells that dysregulates lipid production. Hence, low levels of high-density lipoproteins (HDL) prevent them from binding to and neutralizing pathogen-associated lipids, resulting in chronic inflammation, as occurs in PCC^(15)^. As a result, the association between obesity and PCC causes multisystemic alterations, promoting systemic inflammation that affects organs and muscle tissues^(27)^. Symptoms such as diarrhea are caused by damage to the intestinal epithelial barriers by SARS-CoV-2^(17)^ and due to epithelial damage in blood vessels^(28)^, which explain the symptoms of tingling in the upper limbs.

It should be noted that people with comorbidities, such as DM, SAH, and obesity, have a significantly higher prevalence of being hospitalized due to COVID-19. Comorbidities compromise the immune system and increase vulnerability to serious complications, such as respiratory failure, acute respiratory distress syndrome, and cardiovascular problems. These factors make the infection more difficult to control and increase the need for hospitalization. Furthermore, the presence of comorbidities can prolong hospital stays and increase the risk of long-term sequelae, such as PCC^(24)^.

However, although the presence of comorbidities is considered a risk factor for PCC, the literature has pointed to COVID-19 vaccination as a protective factor against the development of post-COVID disease. An analysis indicated that two-dose vaccination before viral infection was associated with a 24% reduction in the chances of PCC, and vaccination after viral infection with one dose was associated with a 15% reduction in the chances of PCC^(29)^. Therefore, encouraging COVID-19 vaccination in patients with PCC is critical, because in addition to reducing the risk of serious complications in the acute disease, vaccination can improve quality of life by reducing persistent symptoms and helping to decrease the transmission of the virus. Therefore, the vaccine plays an essential role in the management and prevention of PCC^(29,30)^.

This study has limitations, such as underestimating the true prevalence of PCC in the sample, given the quality of the data recorded in the electronic medical records; furthermore, although the results suggest relevant associations between the variables analyzed, it is not possible to infer causality in the cross-sectional design, so the findings should be interpreted with caution regarding their generalization. Nevertheless, the findings of this study show that people with hypertension and obesity may present a higher prevalence of symptoms, respectively weakness and/or loss of muscle strength, diarrhea, and tingling and/or throbbing in the upper limbs. This way, the results of this study contribute significantly to scientific advancement by providing a more detailed understanding of how specific comorbidities influence symptoms and provide valuable information for the development of more targeted treatment strategies. These findings may enhance clinical approaches by focusing on interventions tailored to each patient profile, improving the management and quality of life of individuals affected by PCC.

## CONCLUSION

The results of this study show a higher frequency of PCC symptoms among women, of white ethnicity, with an average age of 46.9 years and comorbidities, the most common being DM, SAH, and obesity. Also noteworthy is that individuals with DM showed a decreased prevalence of developing headaches and sore throats compared to non-diabetics; hypertensive individuals tend to present with weakness and/or loss of muscle strength compared to normotensive individuals, and obese individuals had a higher probability to present with diarrhea and upper limbs tingling and/or throbbing. These results highlight the importance of comorbidities in the symptomatic profile of PCC, suggesting the need for specific strategies for managing these individuals. It is suggested that future studies on the presentation of PCC symptoms in people with comorbidities include variables related to age, sex, ethnicity, adherence to recommended treatment for the pre-existing disease, and status of the COVID-19 vaccination schedule, factors that may influence the prevalence and manifestation of symptoms in PCC.

## Data Availability

All the data supporting the results of this study were published in the article itself.
