# Supplementary material for: Key comorbidities in the development of post-COVID conditions: diabetes mellitus, hypertension, and obesity
Source: Rev Esc Enferm USP. 2026 Aug 21;60:e20250473. doi: 10.1590/1980-220X-REEUSP-2025-0473en (PMC13524457; doi:10.1590/1980-220X-REEUSP-2025-0473en)
Supplement: Translation

# Translation: Comorbidades determinantes no desenvolvimento da condição pós-COVID: diabetes mellitus, hipertensão e obesidade

**DOI:** 10.1590/1980-220X-REEUSP-2025-0473pt

**Authors:** Karina Marques Prediger, Ana Cristina Ribeiro La Scaléa, Sílvia Carla da Silva André Uehara

**Affiliations:** 1 Universidade Federal de São Carlos, Departamento de Enfermagem, São Carlos, SP, Brasil.

**Keywords:** Síndrome de Pós-COVID-19 Aguda, Prevalência, Diabetes Mellitus, Hipertensão, Obesidade

## Abstract

**Objetivo:**

Analisar e comparar os sintomas da condição pós-COVID (CPC) em pacientes com e sem comorbidades crônicas, como diabetes, hipertensão e obesidade.

**Método:**

Trata-se de um estudo transversal e analítico, considerando uma amostra de 350 pacientes com CPC atendidos em serviços da Atenção Primária à Saúde. Os dados foram coletados a partir da verificação de prontuários e analisados por meio do modelo de regressão de Poisson para estimar a razão de prevalência.

**Resultados:**

A prevalência para a manifestação dos sintomas da CPC foi reduzida em 50% para dor de cabeça e em 64% para dor de garganta em pessoas não diabéticas. Pessoas hipertensas apresentaram prevalência duas vezes maior de fraqueza e/ou perda da força muscular em relação aos normotensos. Além disso, diarreia e formigamento/latejamento em membros superiores foi mais prevalente em pessoas obesas.

**Conclusão:**

O estudo apresentou uma relação bidirecional entre CPC e as condições crônicas pré-existentes, fornecendo uma compreensão detalhada sobre como as comorbidades específicas influenciam os sintomas da CPC.

## INTRODUÇÃO

Desde o início da pandemia de COVID-19, foi identificado que pessoas com doenças metabólicas possuem risco aumentado para o desenvolvimento da forma grave da doença e para óbito em relação às pessoas sem comorbidades. Ademais, observou-se que parte dos indivíduos comórbidos infectados pelo SARS-CoV-2 apresentavam quadro mais favorável ao desenvolvimento da condição pós-COVID (CPC)^(1,2,3)^.

A CPC é definida pela Organização Mundial da Saúde (OMS) como sendo uma doença caracterizada por sintomas desenvolvidos em indivíduos com histórico de COVID-19, com início três meses após a doença aguda e que não pode ser justificado por um diagnóstico alternativo. Os sintomas de CPC podem ter início após a fase aguda da doença inicial, flutuar ou recair ao longo do tempo, e os mais comuns são fadiga, falta de ar e dor de cabeça^(4)^.

Na literatura, é possível encontrar outros termos sinônimos de CPC, como COVID longa, “COVID-19 pós-aguda”, “síndrome pós-covid”, “efeitos em longo prazo da covid”, “síndrome de covid-19 pós-aguda”, entre outras denominações. No Brasil, o Ministério da Saúde (MS) adotou o termo “condição pós-COVID” a fim de padronizar os documentos técnicos e orientar os profissionais da saúde sobre a temática^(5)^. De acordo com a Nota Técnica Nº 57/2023 do MS, a CPC caracteriza-se pela presença de sinais e/ou sintomas que se desenvolvem em quatro semanas ou mais após a infecção inicial por SARS-CoV-2 e não podem ser justificadas por um diagnóstico alternativo. Além disso, a condição pós-COVID apresenta potencial para melhora, agravamento ou serem recidivantes ao longo do tempo, podendo evoluir para eventos graves em meses ou anos após a infecção aguda^(6)^.

Estima-se que de 20 a 30% e de 50 a 89% dos pacientes com COVID-19 não hospitalizados e hospitalizados, respectivamente, estão suscetíveis ao desenvolvimento dos sintomas da CPC^(7)^. Sabe-se que pessoas do sexo feminino, idade avançada, condições médicas preexistentes, falta de vacinação contra a COVID-19, número de sintomas durante a fase aguda, carga viral, curso grave durante a fase aguda da doença e uso de ventilação mecânica invasiva são fatores que indicam maior propensão à apresentação de sintomas da CPC^(1,2)^.

Sugere-se que os sintomas em longo prazo após a recuperação da fase aguda da COVID-19 estão associados à capacidade do vírus de desencadear uma resposta inflamatória maciça. Frequentemente, os sintomas da CPC também estão relacionados à gravidade na fase aguda da doença, mas também podem ocorrer após uma manifestação leve da COVID-19, bem como afetar pacientes de todas as idades^(8)^. Além disso, os sintomas da CPC podem, inclusive, agravar os sintomas pré-existentes das comorbidades apresentadas previamente pelos indivíduos^(1)^.

Devido às características dos sintomas da CPC, que são comuns a outras enfermidades, o diagnóstico dessa condição é negligenciado e complexo. Atualmente, o maior desafio no atendimento de pacientes com CPC é o fato de essa doença não poder ser considerada uma entidade clínica única, necessitando de um tratamento multidisciplinar integrado, devido à diversidade de sintomas^(2)^. Assim, compreender o curso dessa doença é determinante na recuperação e promoção da melhora na qualidade de vida das pessoas.

Ainda, a etiologia da CPC precisa ser totalmente compreendida frente aos diversos sintomas e fenótipos. Até o momento, a literatura evidencia que a CPC está associada principalmente ao sexo feminino, idade avançada, vulnerabilidade socioeconômica, estado de vacinação contra COVID-19 e condições clínicas pré-existentes, como diabetes, hipertensão e obesidade^(2,3,8,9)^. Entretanto, ainda são escassos os estudos que comparam a manifestação dos sintomas da CPC entre os indivíduos com e sem comorbidades crônicas, o que dificulta a compreensão da influência dessas condições na persistência dos sintomas. Diante do exposto, este estudo tem como objetivo analisar e comparar os sintomas da condição pós-COVID em pacientes com e sem comorbidades crônicas, como diabetes, hipertensão e obesidade.

## MÉTODO

### Desenho do Estudo

Trata-se de um estudo transversal e analítico, elaborado de acordo com as recomendações do *Strengthening The Reporting of Observational Studies in Epidemiology* (STROBE).

### Local

Este estudo foi realizado no município de São Carlos, localizado no estado de São Paulo. Os dados foram obtidos junto aos serviços de Vigilância em Saúde e ao Departamento de Gestão e Cuidado Ambulatorial, setor responsável pelos serviços da Atenção Primária à Saúde (APS).

### População e Critérios de Seleção

Foi realizado um cálculo amostral considerando todos os casos de COVID-19 diagnosticados na população com idade superior a 18 anos no município, referente ao período de 26/02/2020 a 26/02/2024, por meio de Teste Rápido (TR) ou *Reverse Transcription Polymerase Chain Reaction* (RT-PCR). Na cidade de São Carlos-SP, foram notificados 62.977 casos positivos de COVID-19 em indivíduos acima de 18 anos durante o período de análise deste estudo. A escolha da prevalência baseou-se em dados da literatura^(10)^, que indicam que cerca de 50% dos casos de COVID-19 apresentaram sintomas da CPC. Foram incluídos no estudo pacientes adultos acima de 18 anos que apresentaram teste positivo para COVID-19 e retornaram aos serviços da APS com sintomas da CPC, conforme a definição da OMS, que descreve os sintomas como sendo reincidentes ou flutuantes por um período mínimo de dois meses após a infecção pela doença aguda^(4)^. Foram excluídos do estudo pacientes cujo prontuário não estava acessível, que não residiam em localidades adscritas à APS ou que faleceram. As variáveis do estudo foram sexo, idade, raça/cor, comorbidades, esquema vacinal e os sintomas relatados.

### Cálculo do Tamanho da Amostra

Para o cálculo amostral, assumiu-se um nível de significância de 5% (α = 0,05), erro absoluto tolerável de 5 pontos percentuais (d = 0,05) e uma população finita estimada em N = 31.489 pacientes com CPC acima de 18 anos no município de interesse. A prevalência esperada utilizada foi p = 0,356, correspondente à frequência do sintoma fadiga, uma das manifestações mais relatadas em estudos prévios sobre CPC. Assim, o tamanho amostral adotado neste estudo foi de n = 350. O cálculo foi realizado por meio da seguinte equação:


$$n=\frac{z_{\frac{\left(1-\alpha \right)}{2}}^{2}Np(1-p)}{d^{2}(N-1)+z_{\frac{\left(1-\alpha \right)}{2}}^{2}p(1-p)}$$

Onde p é a prevalência esperada, d é o erro absoluto tolerável, N é o tamanho da população e z é o valor da distribuição normal padrão correspondente ao nível de confiança desejado.

### Coleta de Dados

Para a coleta de dados, foram construídas planilhas no Microsoft Excel, contendo as variáveis de interesse. Inicialmente, foram criadas diferentes abas para a coleta dos dados provenientes dos diferentes bancos utilizados nesta pesquisa, obtidos no SisCOVID, sistema de notificação de COVID-19 do município de São Carlos-SP. Na aba seguinte, foram inseridos os dados referentes à situação vacinal, obtidos no VaciVida, sistema desenvolvido pelo Estado de São Paulo. Por fim, na última aba, foram inseridos os dados referentes aos sintomas da CPC obtidos no Prontuário Eletrônico do Cidadão (PEC). Foram adotadas medidas para minimizar vieses, incluindo o treinamento dos coletores de dados (dois graduandos, dois mestrandos e um doutorando em Ciência da Saúde), a utilização de critérios de inclusão e exclusão previamente definidos e a padronização da coleta de dados por meio de uma planilha estruturada e digitalizada. Além disso, destacam-se a escolha do nível de significância adequado (α = 0,05), ajuste para múltiplas comparações, a aplicação de métodos estatísticos apropriados e tamanho amostral robusto.

Assim, primeiramente, foi realizada a identificação dos casos confirmados de COVID-19 no SisCOVID; posteriormente, consultou-se no PEC se o paciente infectado pelo SARS-CoV-2 apresentou retorno na APS com relato de sintomas da CPC, de acordo com a definição da OMS^(4)^. Por meio do SisCOVID, também foi possível obter informações sobre consultas de usuários com queixas de sintomas respiratórios após a fase aguda da doença; já pelo PEC, verificaram-se os sintomas da CPC referidos pelos usuários, que atingiam os diferentes sistemas orgânicos do corpo. Além disso, os dados referentes à vacinação foram obtidos na página do VaciVida, junto à vigilância epidemiológica municipal.

### Análise e Tratamento dos Dados

Inicialmente, os dados foram descritos por meio de frequências absolutas e percentuais (variáveis qualitativas) e por medidas de centralidade e dispersão, como média, desvio-padrão, mínimo, mediana e máximo (variáveis quantitativas). Para estimar a razão de prevalência bruta dos sintomas da CPC em relação às variáveis de interesse, foi utilizado o modelo de regressão de Poisson com variância robusta^(11)^. Nas análises em que a frequência dos sintomas era nula, foi realizado o teste exato de Fisher. O uso de *Missing Data* foi indicado no rodapé das tabelas para garantir a transparência dos dados apresentados, mantendo-se o número de casos ausentes conforme observados, sem imputação. Todas as análises foram realizadas com o auxílio do software SAS 9.4^(12)^, adotando-se um nível de significância de 5%.

### Aspectos Éticos

Conforme a Resolução nº 510/2016, o projeto foi aprovado pelo Comitê de Ética em Pesquisa da Universidade Federal de São Carlos (CAAE: 67128023.4.0000.5504; CAAE: 74911623.5.0000.5504).

## RESULTADOS

Para a obtenção da amostra de 350 casos da condição pós-COVID, foram analisados 3.778 casos notificados e confirmados de COVID-19, correspondendo a 9,26% dos casos da doença aguda na população adulta do município de São Carlos-SP. Os resultados mostram que 67,1% (235) eram do sexo feminino, 61,6% (215) de cor branca, 48,2% (169) eram comórbidos, sendo 30,3% (106) com Hipertensão Arterial Sistêmica (HAS), 15,1% (53) com Diabetes Mellitus (DM) e 16% (56) com obesidade (16%), e a idade média de 46,9 anos (Tabela 1).

**Tabela 1:** Caracterização demográfica e clínica da amostra do estudo – São Carlos, SP, Brasil, 2024.

Características	Frequência (n = 350)	Percentual (%)
**Sexo**		
Feminino	235	67,1
Masculino	115	32,9
**Idade**		
Média	46,9 [15,8]	–
Mediana (Mínimo e Máximo)	47,0 [14,0 e 88,0]	–
**Faixa etária**		
< 60 anos	270	77,1%
≥ 60 anos	80	22,9%
**Raça/Cor**		
Amarela	1	0,3
Branco	215	61,6
Pardo	105	30,1
Preto	28	8,0
Missing	1	–
**Comorbidades**		
Hemoglobinopatias	1	0,3
Cirrose hepática	0	0
Doenças cardiovasculares	17	4,9
Doenças neurológicas	4	1,1
Diabetes Mellitus (Tipo 1 e Tipo 2)	53	15,1
Doença Renal Crônica	4	1,1
Hipertensão Arterial Sistêmica	106	30,3
Imunossuprimidos	0	0
Obesidade	56	16
Pneumopatias crônicas graves	21	6,0
Outros	51	14,6

A prevalência dos sintomas da CPC, considerando a ausência ou a presença de comorbidades, foi reduzida em 58% para a manifestação de coriza (IC95% 0,23;0,78), em 31% para dor de cabeça (IC95% 0,49;0,98) e em 46% para dor de garganta (IC95% 0,34;0,85) em pessoas sem comorbidades (Tabela 2).

**Tabela 2:** Comparação da presença de sintomas da condição pós-COVID, considerando a presença de comorbidades na amostra do estudo – São Carlos, SP, Brasil, 2024.

Descrição dos sintomas da condição pós-COVID	Não Comórbido (n = 181)	Comórbido (n = 169)	Razão de prevalência [IC 95%]*	Valor p^†^
Alteração na menstruação^‡^	6 (3,6%)	2 (1,2%)	0,34 (0,07; 1,68)	0,19
Ansiedade^‡^	11 (6,6%)	10 (6,2%)	0,94 (0,41; 2,15)	0,88
Cansaço	32 (17,1%)	25 (14,8%)	0,84 (0,52; 1,35)	0,47
Coriza	33 (18,2%)	13 (7,7%)	0,42 (0,23; 0,78)	<0,01
Diarreia	1 (0,6%)	3 (1,8%)	3,21 (0,34; 30,59)	0,31
Dificuldade de concentração^‡^	3 (1,8%)	2 (1,2%)	0,69 (0,12; 4,06)	0,68
Dor abdominal^‡^	12 (7,2%)	9 (5,6%)	0,77 (0,33; 1,79)	0,55
Dor articular^‡^	4 (2,4%)	3 (1,9%)	0,77 (0,18; 3,4)	0,73
Dor de cabeça	59 (32,6%)	38 (22,5%)	0,69 (0,49; 0,98)	0,04
Dor de garganta	44 (24,3%)	22 (13,0%)	0,54 (0,34; 0,85)	<0,01
Dor em MMII^‡^	14 (8,4%)	25 (15,4%)	1,84 (0,99; 3,41)	0,05
Dor em MMSS^‡^	6 (3,6%)	9 (5,6%)	1,55 (0,56; 4,25)	0,40
Dor muscular	28 (15,5%)	23 (13,6%)	0,88 (0,53; 1,47)	0,62
Dor nas costas/lombar^‡^	19 (11,4%)	11 (6,8%)	0,6 (0,29; 1,21)	0,15
Dor no ouvido^‡^	0 (0%)	1 (0,6%)	-	0,49^§^
Dor no peito^‡^	8 (4,8%)	9 (5,6%)	1,16 (0,46; 2,93)	0,75
Dor no tórax^‡^	8 (4,8%)	5 (3,1%)	0,64 (0,22; 1,93)	0,43
Falta de ar	22 (12,2%)	34 (20,1%)	1,66 (1,01; 2,71)	0,05
Formigamento/latejamento em MMII^‡^	7 (4,2%)	9 (5,6%)	1,33 (0,51; 3,47)	0,57
Formigamento/latejamento em MMSS^‡^	6 (3,6%)	9 (5,6%)	1,55 (0,56; 4,25)	0,40
Fraqueza/perda da força muscular^‡^	12 (7,2%)	18 (11,1%)	1,55 (0,77; 3,11)	0,22
Insônia^‡^	9 (5,4%)	10 (6,2%)	1,15 (0,48; 2,75)	0,76
Febre	19 (10,5%)	15 (8,9%)	0,85 (0,44; 1,61)	0,61
Náusea	6 (3,3%)	2 (1,2%)	0,36 (0,07; 1,74)	0,20
Odinofagia^‡^	2 (1,2%)	0 (0%)	-	0,50^§^
Perda de memória/confusão^‡^	5 (3,0%)	10 (6,2%)	2,06 (0,72; 5,9)	0,18
Perda de olfato	5 (2,8%)	2 (1,2%)	0,43 (0,08; 2,18)	0,31
Perda de paladar	3 (1,7%)	1 (0,6%)	0,36 (0,04; 3,4)	0,37
Queda de cabelo^‡^	19 (11,4%)	11 (6,8%)	0,6 (0,29; 1,21)	0,15
Queixa odontológica de perda dentária pós-COVID^‡^	2 (1,2%)	1 (0,6%)	0,52 (0,05; 5,63)	0,59
Tristeza/depressão^‡^	5 (3,0%)	10 (6,2%)	2,06 (0,72; 5,9)	0,18
Tontura^‡^	7 (4,2%)	8 (4,9%)	1,18 (0,44; 3,17)	0,75
Tosse	44 (24,3%)	42 (24,9%)	1,02 (0,71; 1,48)	0,91
Vômito	9 (5,0%)	3 (1,8%)	0,36 (0,1; 1,3)	0,12

*IC = Intervalo de Confiança;

^†^Valor p = Nível de significância;

^‡^Missing 20;

^§^Valor referente ao teste exato de Fisher.

Dentre as comorbidades mais frequentes na população de estudo, foram analisadas a relação entre a presença de sintomas da CPC e HAS, DM e obesidade, com maior destaque à primeira. Assim, em relação à DM, destaca-se que a ansiedade foi referida apenas na amostra que não apresentava essa comorbidade. Já a prevalência para a manifestação dos sintomas da CPC foi reduzida em 50% para dor de cabeça (IC95% 0,26;0,98) e em 64% para dor de garganta (IC95% 0,14;0,95) em pessoas não diabéticas (Tabela 3).

**Tabela 3:** Comparação da presença de sintomas da condição pós-COVID considerando o diagnóstico de DM da população de estudo – São Carlos, SP, Brasil, 2024.

Descrição dos sintomas da condição pós-COVID	Não diabéticos [A] (n = 297)	Diabéticos [B] (n = 53)	Razão de prevalência [B/A] [IC 95%]*	Valor p^†^
Alteração na menstruação^‡^	8 (2,9%)	0 (0%)	–	0,36^§^
Ansiedade^‡^	21 (7,6%)	0 (0%)	–	0,03^§^
Cansaço	49 (16,5%)	8 (15,1%)	0,91 (0,46; 1,82)	0,80
Coriza	44 (14,9%)	2 (3,8%)	0,25 (0,06; 1,02)	0,05
Diarreia	2 (0,7%)	2 (3,8%)	5,6 (0,81; 38,92)	0,08
Dificuldade de concentração^‡^	5 (1,8%)	0 (0%)	–	0,99^§^
Dor abdominal^‡^	17 (6,2%)	4 (7,5%)	1,23 (0,43; 3,5)	0,70
Dor articular^‡^	5 (1,8%)	2 (3,8%)	2,08 (0,42; 10,46)	0,37
Dor de cabeça	89 (30,0%)	8 (15,1%)	0,5 (0,26; 0,98)	0,04
Dor de garganta	62 (20,9%)	4 (7,5%)	0,36 (0,14; 0,95)	0,04
Dor em MMII^‡^	33 (12,0%)	6 (11,3%)	0,95 (0,42; 2,15)	0,90
Dor em MMSS^‡^	10 (3,6%)	5 (9,4%)	2,6 (0,93; 7,31)	0,07
Dor muscular	46 (15,5%)	5 (9,4%)	0,61 (0,25; 1,46)	0,27
Dor nas costas/lombar^‡^	25 (9,1%)	5 (9,4%)	1,04 (0,42; 2,6)	0,93
Dor no ouvido^‡^	0 (0%)	1 (1,9%)	–	0,16^§^
Dor no peito^‡^	15 (5,4%)	2 (3,8%)	0,69 (0,16; 2,95)	0,62
Dor no tórax^‡^	12 (4,3%)	1 (1,9%)	0,43 (0,06; 3,27)	0,42
Falta de ar	44 (14,8%)	12 (22,6%)	1,53 (0,87; 2,7)	0,14
Formigamento/latejamento em MMII^‡^	11 (4,0%)	5 (9,4%)	2,37 (0,86; 6,53)	0,10
Formigamento/latejamento em MMSS^‡^	7 (2,5%)	4 (7,5%)	2,98 (0,9; 9,81)	0,07
Fraqueza/perda da força muscular^‡^	22 (8,0%)	8 (15,1%)	1,89 (0,89; 4,02)	0,10
Insônia^‡^	17 (6,2%)	2 (3,8%)	0,61 (0,15; 2,57)	0,50
Febre	32 (10,8%)	2 (3,8%)	0,35 (0,09; 1,42)	0,14
Náusea	8 (2,7%)	0 (0%)	–	0,61^§^
Odinofagia^‡^	2 (0,7%)	0 (0%)	–	0,99^§^
Perda de memória/confusão^‡^	12 (4,3%)	3 (5,7%)	1,3 (0,38; 4,46)	0,67
Perda de olfato	7 (2,4%)	0 (0%)	–	0,60^§^
Perda de paladar	4 (1,3%)	0 (0%)	–	0,99^§^
Queda de cabelo^‡^	25 (9,1%)	5 (9,4%)	1,04 (0,42; 2,6)	0,93
Queixa odontológica de perda dentária pós-COVID^‡^	3 (1,1%)	0 (0%)	–	0,99^§^
Tristeza/depressão^‡^	13 (4,7%)	2 (3,8%)	0,8 (0,19; 3,45)	0,77
Tontura^‡^	12 (4,3%)	3 (5,7%)	1,3 (0,38; 4,46)	0,67
Tosse	75 (25,3%)	11 (20,8%)	0,82 (0,47; 1,44)	0,49
Vômito	11 (3,7%)	1 (1,9%)	0,51 (0,07; 3,86)	0,51

*IC = Intervalo de Confiança;

^†^Valor p = Nível de significância;

^‡^Missing 20;

^§^Valor referente ao teste exato de Fisher.

Os resultados mostram que pessoas hipertensas apresentaram prevalência duas vezes maior de manifestar fraqueza e/ou perda da força muscular em relação aos normotensos (IC95% 1,13;4,38); ainda, hipertensos não referiram a presença de náusea, odinofagia e vômitos (Tabela 4).

**Tabela 4:** Comparação da presença de sintomas da condição pós-COVID considerando o diagnóstico de HAS da população de estudo – São Carlos, SP, Brasil, 2024.

Descrição dos sintomas da condição pós-COVID	Normotensos [A] (n = 244)	Hipertensos [B] (n = 106)	Razão de prevalência [B/A] [IC 95%]*	Valor p^†^
Alteração na menstruação^‡^	7 (3,1%)	1 (1,0%)	0,32 (0,04; 2,55)	0,28
Ansiedade^‡^	15 (6,6%)	6 (5,9%)	0,89 (0,36; 2,23)	0,80
Cansaço	40 (16,4%)	17 (16,0%)	0,98 (0,58; 1,64)	0,93
Coriza	34 (14,0%)	12 (11,3%)	0,81 (0,44; 1,5)	0,50
Diarreia	3 (1,2%)	1 (0,9%)	0,77 (0,08; 7,29)	0,82
Dificuldade de concentração^‡^	4 (1,8%)	1 (1,0%)	0,56 (0,06; 4,92)	0,60
Dor abdominal^‡^	13 (5,7%)	8 (7,8%)	1,37 (0,59; 3,2)	0,47
Dor articular^‡^	5 (2,2%)	2 (2,0%)	0,89 (0,18; 4,51)	0,89
Dor de cabeça	71 (29,1%)	26 (24,5%)	0,84 (0,57; 1,24)	0,39
Dor de garganta	51 (20,9%)	15 (14,2%)	0,68 (0,1; 1,15)	0,15
Dor em MMII^‡^	22 (9,7%)	17 (16,7%)	1,72 (0,96; 3,1)	0,07
Dor em MMSS^‡^	10 (4,4%)	5 (4,9%)	1,11 (0,39; 3,17)	0,84
Dor muscular	36 (14,8%)	15 (14,2%)	0,96 (0,55; 1,67)	0,88
Dor nas costas/lombar^‡^	23 (10,1%)	7 (6,9%)	0,68 (0,3; 1,53)	0,35
Dor no ouvido^‡^	0 (0%)	1 (1,0%)	–	0,31^§^
Dor no peito^‡^	13 (5,7%)	4 (3,9%)	0,68 (0,23; 2,05)	0,50
Dor no tórax^‡^	12 (5,3%)	1 (1,0%)	0,19 (0,02; 1,41)	0,10
Falta de ar	35 (14,3%)	21 (19,8%)	1,38 (0,85; 2,26)	0,20
Formigamento/latejamento em MMII^‡^	11 (4,8%)	5 (4,9%)	1,01 (0,36; 2,84)	0,98
Formigamento/latejamento em MMSS^‡^	8 (3,5%)	3 (2,9%)	0,83 (0,23; 3,08)	0,79
Fraqueza/perda da força muscular^‡^	15 (6,6%)	15 (14,7%)	2,23 (1,13; 4,38)	0,02
Insônia^‡^	11 (4,8%)	8 (7,8%)	1,62 (0,67; 3,9)	0,28
Febre	21 (8,6%)	13 (12,3%)	1,43 (0,74; 2,74)	0,29
Náusea	8 (3,3%)	0 (0%)	–	0,11^§^
Odinofagia^‡^	2 (0,9%)	0 (0%)	–	0,99^§^
Perda de memória/confusão^‡^	8 (3,5%)	7 (6,9%)	1,95 (0,73; 5,23)	0,19
Perda de olfato	5 (2,0%)	2 (1,9%)	0,92 (0,18; 4,67)	0,92
Perda de paladar	3 (1,2%)	1 (0,9%)	0,77 (0,08; 7,29)	0,82
Queda de cabelo^‡^	25 (11,0%)	5 (4,9%)	0,45 (0,18; 1,13)	0,09
Queixa odontológica de perda dentária pós-COVID^‡^	2 (0,9%)	1 (1,0%)	1,11 (0,1; 12,13)	0,93
Tristeza/depressão^‡^	8 (3,5%)	7 (6,9%)	1,95 (0,73; 5,23)	0,19
Tontura^‡^	10 (4,4%)	5 (4,9%)	0,45 (0,18; 1,13)	0,09
Tosse	55 (22,5%)	31 (29,2%)	1,3 (0,89; 1,89)	0,18
Vômito	12 (4,9%)	0 (0%)	–	0,02^§^

*IC = Intervalo de Confiança;

^†^Valor p = Nível de significância;

^‡^Missing 20;

^§^Valor referente ao teste exato de Fisher.

Pessoas com obesidade não relataram a presença de coriza, dor de ouvido, insônia, perda de olfato e paladar, queixa odontológica e vômito. Além disso, diarreia (IC95% 1,67;148,7) e formigamento/latejamento em membros superiores (IC95% 1,89;18,9) foi mais prevalente em pessoas obesas. Por outro lado, a prevalência dos sintomas da CPC em pessoas obesas foi reduzida em 59% para manifestação de dor de cabeça (IC95% 0,2;0,83) e em 84% para dor de garganta (IC95% 0,04;0,65) em relação aos eutróficos (Tabela 5).

**Tabela 5:** Comparação da presença de sintomas da condição pós-COVID, considerando o diagnóstico de obesidade da amostra do estudo – São Carlos, SP, Brasil, 2024.

Descrição dos sintomas da condição pós-COVID	Eutróficos [A] (n = 294)	Obesos [B] (n = 56)	Razão de prevalência [B/A] [IC 95%]*	Valor p^†^
Alteração na menstruação^‡^	7 (2,6%)	1 (1,8%)	0,71 (0,09; 5,67)	0,75
Ansiedade^‡^	15 (5,5%)	6 (109%)	1,99 (0,81; 4,91)	0,13
Cansaço	51 (17,3)	6 (10,7%)	0,62 (0,28; 1,37)	0,24
Coriza	46 (15,7%)	0 (0%)	–	<0,01^§^
Diarreia	1 (0,3%)	3 (5,4%)	15,75 (1,67; 148,7)	0,02
Dificuldade de concentração^‡^	4 (1,5%)	1 (1,8%)	1,25 (0,14; 0,93)	0,84
Dor abdominal^‡^	20 (7,3%)	1 (1,8%)	0,25 (0,03; 1,82)	0,17
Dor articular^‡^	6 (2,2%)	1 (1,8%)	0,83 (0,1; 6,76)	0,86
Dor de cabeça	90 (30,6%)	7 (1,5%)	0,41 (0,2; 0,83)	0,01
Dor de garganta	64 (21,8%)	2 (3,6%)	0,16 (0,04; 0,65)	0,01
Dor em MMII^‡^	29 (10,6%)	10 (18,2%)	1,72 (0,89; 3,32)	0,11
Dor em MMSS^‡^	11 (4,0%)	4 (7,3%)	1,81 (0,6; 5,48)	0,29
Dor muscular	47 (16,0%)	4 (7,1%)	0,45 (0,17; 1,19)	0,11
Dor nas costas/lombar^‡^	26 (9,5%)	4 (7,3%)	0,77 (0,28; 2,11)	0,61
Dor no ouvido^‡^	1 (0,4%)	0 (0%)	–	0,99^§^
Dor no peito^‡^	16 (5,8%)	1 (1,8%)	0,31 (0,04; 2,3)	0,25
Dor no tórax^‡^	12 (4,4%)	1 (1,8%)	0,42 (0,06; 3,13)	0,39
Falta de ar	51 (17,3%)	5 (8,9%)	0,51 (0,22; 1,23)	0,14
Formigamento/latejamento em MMII^‡^	12 (4,4%)	4 (7,3%)	1,66 (0,56; 4,96)	0,36
Formigamento/latejamento em MMSS^‡^	5 (1,8%)	6 (10,9%)	5,98 (1,89; 18,9)	<0,01
Fraqueza/perda da força muscular^‡^	25 (9,1%)	5 (9,1%)	1 (0,4; 2,49)	0,99
Insônia^‡^	19 (6,9%)	0 (0%)	–	0,05^§^
Febre	33 (11,2%)	1 (1,8%)	0,16 (0,02; 1,14)	0,07
Náusea	6 (2,0%)	2 (3,6%)	1,75 (0,36; 8,45)	0,49
Odinofagia^‡^	2 (0,7%)	0 (0%)	–	0,99^§^
Perda de memória/confusão^‡^	11 (4,0%)	4 (7,3%)	1,81 (0,6; 5,48)	0,29
Perda de olfato	7 (2,4%)	0 (0%)	–	0,60^§^
Perda de paladar	4 (1,4%)	0 (0%)	–	0,99^§^
Queda de cabelo^‡^	25 (9,1%)	5 (9,1%)	1 (0,4; 2,49)	0,99
Queixa odontológica de perda dentária pós-COVID^‡^	3 (1,1%)	0 (0%)	–	0,99^§^
Tristeza/depressão^‡^	12 (4,4%)	3 (5,5%)	1,25 (0,36; 4,27)	0,73
Tontura^‡^	13 (4,7%)	2 (3,6%)	0,77 (0,18; 3,3)	0,72
Tosse	78 (26,5%)	8 (14,3%)	0,54 (0,28; 1,05)	0,07
Vômito	12 (4,1%)	0 (0%)	–	0,23^§^

*IC = Intervalo de Confiança;

^†^Valor p = Nível de significância;

^‡^Missing 20;

^§^Valor referente ao teste exato de Fisher.

## DISCUSSÃO

Esta análise evidenciou que indivíduos sem DM apresentaram prevalência reduzida para a manifestação de sintomas como dor de cabeça e dor de garganta, enquanto hipertensos mostraram maior propensão à fraqueza muscular. Já pessoas obesas tiveram maior prevalência ao desenvolvimento de diarreia e formigamento nos membros superiores, mas menor prevalência para dor de cabeça e garganta. Esses resultados destacam a influência das comorbidades nos sintomas da CPC, sugerindo que a presença de DM, HAS e obesidade pode modificar o perfil sintomatológico de acordo com cada condição. Assim como os resultados deste estudo, a literatura também destaca que as comorbidades mais frequentes em indivíduos com CPC são DM, HAS e obesidade^(13)^.

Nesta conjuntura, buscando esclarecer as relações estabelecidas entre a presença de comorbidades, como DM, HAS e obesidade, com a ocorrência dos sintomas da CPC, sugere-se uma relação com o processo inflamatório crônico pós infecção pelo SARS-CoV-2. Além da prevalência aumentada de pessoas com DM apresentarem agravamento na COVID-19, esse grupo é mais frequentemente acometido pela CPC, sendo mais suscetíveis às consequências em longo prazo do que pessoas sem DM. Sabe-se que pacientes com DM apresentam uma inflamação crônica devido à sinalização de insulina prejudicada, ocasionando uma diminuição nas citocinas anti-inflamatórias e maior expressão das citocinas pró-inflamatórias, como fator de necrose tumoral alfa (TNF-α), interleucina 6 (IL-6) e interleucina beta (IL-1β). Assim, com a atividade das citocinas, ocorre a inibição da sinalização de insulina; consequentemente, há um aumento da resistência à insulina, promovendo insuficiência respiratória e colapso cardiopulmonar^(3)^.

É notório que a pandemia da COVID-19 influenciou o controle da DM, bem como a progressão da pré-diabetes e aumento do número de casos da doença em virtude do uso de corticosteroides, devido à terapia em altas doses que resultou em hiperglicemia grave^(1,14,15)^. Agonistas de medicações utilizadas para controle glicêmico, como Pioglitazona, aumentam a secreção do gene ECA 2, havendo indícios de que essa medicação inibe a secreção de citocinas pró-inflamatórias e aumenta as anti-inflamatórias, além de atenuar a lesão pulmonar e reduzir a reação fibrótica pulmonar. Ainda, pessoas diabéticas e usuárias de Metformina têm a probabilidade de reduzir infecções por COVID-19^(3,16,17)^ e, consequentemente, pode influenciar na ausência de desenvolvimento dos sintomas da CPC.

Um estudo no Reino Unido mostrou que pessoas com DM (tipo 1 ou 2) foram associadas a uma maior probabilidade de um diagnóstico registrado da CPC. Ao analisar a prevalência da CPC entre homens com DM2 e seus controles pareados, a prevalência foi menor em controles pareados em 0,46% vs 0,54% vs homens sem DM2 (p = 0,008). Além disso, o estudo supracitado apontou que a prevalência da CPC foi de 0,54% em homens com DM2 e de 0,53% em mulheres com DM2, sem diferença estatisticamente significativa entre os gêneros. Uma hipótese para explicar a semelhança na prevalência entre homens e mulheres com DM e sintomas da CPC poderia estar relacionada ao acompanhamento regular de sua condição crônica, o que os torna potencialmente mais propensos a relatar sintomas, incluindo os relacionados à CPC, aumentando, assim, a probabilidade de detecção dessa condição^(18)^.

Neste estudo, pessoas sem DM apresentaram prevalências diminuídas para o desenvolvimento de dor de cabeça e dor de garganta como sintomas da CPC. A fisiopatologia da dor na CPC é multifatorial e envolve vários mecanismos. A dor persistente em pacientes com CPC pode ser explicada por fatores como inflamação sistêmica e no sistema nervoso central, que sensibilizam os receptores de dor, além de distúrbios na perfusão cerebral e danos neuronais diretos causados pelo vírus. Alterações nos neurotransmissores, como serotonina e dopamina, e fatores psicológicos, como estresse e ansiedade, também agravam a dor. Esses mecanismos, isolados ou combinados, contribuem para as dores persistentes^(19)^.

Nos Estados Unidos, uma análise com pessoas com DM que tiveram COVID-19 indicou que o controle glicêmico inadequado e hemoglobina glicada elevada (HbAlc≥8%) aumentam o risco de surgimento de novos sintomas recorrentes da CPC, especialmente sintomas respiratórios e confusão mental, entre 30 e 180 dias após a infeção por COVID-19, sugerindo sequelas persistentes associadas a infecções agudas. Contudo, os mecanismos para tais achados são hipotéticos, apontando para a redução do estresse oxidativo e inflamação; consequentemente, o tratamento da doença crônica contribuiria na redução dos riscos de desenvolver a CPC^(20)^.

No que se refere à análise da hipertensão e a CPC, neste estudo, pessoas hipertensas apresentaram maior prevalência de desenvolverem sintomas de fraqueza e/ou perda da força muscular em relação ao normotensos. Um estudo realizado na Espanha mostrou que a hipertensão não foi associada a nenhum sintoma específico da CPC; no entanto, uma proporção maior de pacientes hipertensos relatou ≥ 3 sintomas da CPC em comparação aos pacientes normotensos^(21)^. As manifestações musculoesqueléticas associadas à CPC incluem mialgia, dor nas articulações e fraqueza muscular. Pacientes que fizeram uso de corticosteroides durante a fase aguda da doença e pessoas internadas em unidades de terapia intensiva (UTI) podem desenvolver fraqueza muscular até um ano após a infecção. A fraqueza muscular também foi observada em indivíduos com quadro inicial leve de COVID-19, embora não se tenha esclarecido se isso é resultado apenas de descondicionamento ou desuso muscular^(22)^.

Os mecanismos subjacentes à fraqueza muscular ainda não são completamente compreendidos, mas algumas explicações possíveis incluem danos diretos aos neurônios motores e aos músculos adjacentes causados pelo vírus ou pela resposta imunológica durante a infecção aguda por COVID-19. Além disso, o vírus pode prejudicar as mitocôndrias e interferir na cadeia de transporte de elétrons, que é responsável pela produção de energia nos músculos. Esse processo contribui para a fatigabilidade, fraqueza e redução da tolerância ao exercício^(23)^.

No contexto da hospitalização decorrente de COVID-19, a permanência prolongada na UTI foi associada à perda substancial de massa muscular, com uma redução média de 18,5% no volume do músculo reto femoral até o 7º dia de internação. Também se aponta que a sedação profunda, essencial para a implementação da Ventilação Mecânica Invasiva (VMI), compromete a função cardiopulmonar e induz um estado de imobilidade total, o que afeta negativamente a musculatura esquelética. Quando a sedação sozinha não é suficiente para garantir a VMI, bloqueadores neuromusculares são usados para sincronizar o paciente com o ventilador, mas esses agentes interrompem a transmissão de impulsos nas células musculares. A persistência do bloqueio neuromuscular pode resultar em paralisia prolongada, miopatia e fraqueza muscular generalizada^(24)^. Deste modo, as sequelas musculares observadas na CPC decorrem de uma disfunção muscular persistente que se inicia na fase aguda da doença, principalmente devido à diminuição da síntese proteica muscular, agravada por um estado hiperinflamatório, hipoxemia, infecção das células musculares e desregulação do Sistema Renina-Angiotensina (SRA).

Já no que diz respeito às pessoas obesas, neste estudo os obesos apresentaram maior prevalência para o desenvolvimento de diarreia e para formigamento e/ou latejamento em membros superiores. Foi constatado que pacientes com Índice de Massa Corporal (IMC) basal na faixa do sobrepeso ou obesidade possuíam risco aumentado de apresentarem sintomas da CPC, sendo que aqueles com IMC maior que 30 kg/m^2^ portavam um risco aumentado em 10% de relatar sintomas da CPC em relação aos pacientes que possuíam um IMC compreendido no intervalo entre 18,5 e 25 kg/m^2(25)^.

Igualmente, a obesidade caracteriza-se pelo desenvolvimento do estado inflamatório crônico, considerado um componente-chave na resistência à insulina. Assim, a expansão do tecido adiposo ocorre em decorrência da sobrecarga calórica e do aumento na infiltração de células imunes, promovendo uma resposta pró-inflamatória subsequente por meio do aumento de TNF-α, IL1β ou IL-6. Além disso, a ativação do receptor Toll-Like (TLR) em razão da infecção por patógenos, como o SARS-CoV-2, induz à inflamação, expressando-se no músculo esquelético e no tecido adiposo^(26)^.

Como o SARS-CoV-2 é um vírus envelopado, ou seja, uma bicamada lipídica o envolve, ele desempenha um papel importante no ciclo de vida viral pelo seu metabolismo lipídico. Assim, o vírus utiliza o envelope lipídico para promover a invasão ao hospedeiro, objetivando a síntese lipídica e a modificação do sinal das células hospedeiras que culminam na “tempestade de citocinas” devido à ativação excessiva de células imunes que desregula a produção de lipídios. Desse modo, as lipoproteínas de alta densidade (HDL) em baixa quantidade são impedidas de ligar-se e neutralizar lipídios associados a patógenos que resultam na inflamação crônica, como acontece na CPC^(15)^. Assim, a associação entre obesidade e a CPC causa alterações multissistêmicas, promovendo uma inflamação sistêmica que atinge os órgãos e tecidos musculares^(27)^. Sintomas como diarreia são ocasionados pelos danos nas barreiras epiteliais intestinais ocasionadas pelo SARS-CoV-2^(17)^ e pelos danos epiteliais nos vasos sanguíneos^(28)^ que justificam os sintomas de formigamento em membros superiores.

Destaca-se que pessoas com comorbidades, como DM, HAS e obesidade têm uma prevalência significativamente maior de serem hospitalizadas devido à COVID-19. As comorbidades comprometem o sistema imunológico e aumentam a vulnerabilidade a complicações graves, como insuficiência respiratória, síndrome do desconforto respiratório agudo e problemas cardiovasculares. Esses fatores tornam a infecção mais difícil de ser controlada e aumentam a necessidade de hospitalização. Além disso, a presença de comorbidades pode prolongar o tempo de internação e aumentar o risco de sequelas em longo prazo, como a CPC^(24)^.

Contudo, embora a presença de comorbidades seja considerada como um fator de risco para a CPC, a literatura tem apontado a vacinação contra a COVID-19 como um fator protetor para o desenvolvimento da doença pós-COVID. Uma análise apontou que a vacinação antes da infecção viral com duas doses foi associada a uma redução de 24% nas chances da CPC e a vacinação após a infecção viral com uma dose foi associada a uma redução de 15% nas chances da CPC^(29)^. Deste modo, incentivar a vacinação contra a COVID-19 em pacientes com CPC é crucial, pois além de a vacinação diminuir o risco de complicações graves na doença aguda, pode melhorar a qualidade de vida ao reduzir sintomas persistentes e ajudar na diminuição da transmissão do vírus. Portanto, a vacina desempenha um papel essencial na gestão e prevenção da CPC^(29,30)^.

Este estudo apresenta limitações, como subestimar a verdadeira prevalência da CPC na amostra, em vista da qualidade dos dados registrados nos prontuários eletrônicos; além disso, embora os resultados encontrados sugiram associações relevantes entre as variáveis analisadas, não é possível inferir causalidade no delineamento transversal, de modo que os achados devem ser interpretados com cautela quanto à sua generalização. Entretanto, os achados deste estudo evidenciam que pessoas com HAS e obesas podem apresentar maior prevalência de sintomas, respectivamente fraqueza e/ou perda da força muscular, diarreia e formigamento e/ou latejamento em membros superiores. Deste modo, os resultados deste estudo contribuem significativamente para o avanço científico ao fornecer uma compreensão mais detalhada sobre como as comorbidades específicas influenciam os sintomas e fornecem informações valiosas para o desenvolvimento de estratégias de tratamento mais direcionadas. Esses achados podem aprimorar as abordagens clínicas, focando em intervenções adaptadas a cada perfil de paciente, melhorando a gestão e a qualidade de vida dos indivíduos afetados pela CPC.

## CONCLUSÃO

Os resultados deste estudo mostram uma maior frequência de sintomas da CPC entre pessoas do sexo feminino, de cor branca e com idade média de 46,9 anos e com comorbidades, sendo as mais comuns a DM, HAS e obesidade. Também, destaca-se que indivíduos com DM apresentaram prevalência diminuída para o desenvolvimento de dor de cabeça e dor de garganta em relação aos não diabéticos; hipertensos tendem a apresentar fraqueza e/ou perda da força muscular em relação aos normotensos e obesos tiveram maior probabilidade de apresentar diarreia e formigamento e/ou latejamento em membros superiores. Esses resultados destacam a importância das comorbidades no perfil sintomatológico da CPC, sugerindo a necessidade de estratégias específicas para o manejo desses indivíduos. Sugere-se que estudos futuros sobre a apresentação de sintomas da CPC em pessoas com comorbidades incluam variáveis relacionadas a idade, sexo, étnica, adesão ao tratamento recomendado para a doença preexistente e situação do esquema vacinal contra a COVID-19, fatores esses que podem influenciar a prevalência e manifestação dos sintomas na CPC.

## Data Availability

Todo o conjunto de dados que dá suporte aos resultados deste estudo foi publicado no próprio artigo.

# Translation: Comorbilidades determinantes en el desarrollo de condiciones pos-COVID: diabetes mellitus, hipertensión y obesidad

**DOI:** 10.1590/1980-220X-REEUSP-2025-0473es

**Authors:** Karina Marques Prediger, Ana Cristina Ribeiro La Scaléa, Sílvia Carla da Silva André Uehara

**Affiliations:** 1 Universidade Federal de São Carlos, Departamento de Enfermagem, São Carlos, SP, Brasil.

**Keywords:** Síndrome Post Agudo de COVID-19, Prevalencia, Diabetes Mellitus, Hipertensión, Obesidad

## Abstract

**Objetivo:**

Analizar y comparar los síntomas de la afección post-COVID (APC) en pacientes con y sin comorbilidades crónicas, como diabetes, hipertensión y obesidad.

**Método:**

Se trata de un estudio transversal y analítico, que considera una muestra de 350 pacientes con APC tratados en servicios de atención primaria de salud. Los datos se recopilaron a partir de historiales médicos y se analizaron mediante un modelo de regresión de Poisson para estimar la razón de prevalencia.

**Resultados:**

La prevalencia de los síntomas de la APC se redujo en un 50% para el dolor de cabeza y en un 64% para el dolor de garganta en personas no diabéticas. Las personas con hipertensión presentaban el doble de prevalencia de debilidad muscular y/o pérdida de fuerza en comparación con aquellas con presión arterial normal. Además, la diarrea y el hormigueo o palpitaciones en las extremidades superiores fueron más frecuentes en las personas obesas.

**Conclusión:**

El estudio presentó una relación bidireccional entre la APC y las afecciones crónicas preexistentes, lo que permitió comprender en detalle cómo las comorbilidades específicas influyen en los síntomas de la APC.

## INTRODUCCIÓN

Desde el inicio de la pandemia de COVID-19, se ha identificado que las personas con enfermedades metabólicas tienen un mayor riesgo de desarrollar la forma grave de la enfermedad y de fallecer en comparación con las personas sin comorbilidades. Además, se observó que parte de los individuos con comorbilidades infectados por el SARS-CoV-2 presentaban un cuadro más propicio para el desarrollo de las condiciones pos-COVID (CPC)^(1,2,3)^.

La CPC es definida por la Organización Mundial de la Salud (OMS) como una enfermedad caracterizada por síntomas que se presentan en personas con antecedentes de COVID-19, con inicio tres meses después de la fase aguda de la enfermedad y que no pueden explicarse mediante un diagnóstico alternativo. Los síntomas de la CPC pueden aparecer después de la fase aguda de la enfermedad inicial, fluctuar o recaer con el tiempo, y los más comunes son fatiga, dificultad para respirar y dolor de cabeza^(4)^.

En la literatura se pueden encontrar otros términos sinónimos de CPC, como “COVID prolongado”, “COVID-19 posagudo”, “condición pos-COVID”, “efectos a largo plazo del COVID”, “síndrome de COVID-19 posagudo”, entre otras denominaciones. En Brasil, el Ministerio de Salud (MS) adoptó el término “condición pos-COVID” con el fin de estandarizar los documentos técnicos y orientar a los profesionales de la salud sobre el tema^(5)^. De acuerdo con la Nota Técnica N.º 57/2023 del MS, la CPC se caracteriza por la presencia de signos y/o síntomas que se desarrollan cuatro semanas o más después de la infección inicial por el SARS-CoV-2 y que no pueden explicarse mediante un diagnóstico alternativo. Además, la condición pos-COVID tiene el potencial de mejorar, agravarse o recidivar con el tiempo, y puede evolucionar hacia eventos graves meses o años después de la infección aguda^(6)^.

Se estima que entre el 20 % y el 30 % de los pacientes con COVID-19 no hospitalizados, y entre el 50 % y el 89 % de los hospitalizados, respectivamente, son susceptibles de desarrollar los síntomas del CPC^(7)^. Se sabe que el sexo femenino, la edad avanzada, las afecciones médicas preexistentes, la falta de vacunación contra la COVID-19, el número de síntomas durante la fase aguda, la carga viral, el curso grave durante la fase aguda de la enfermedad y el uso de ventilación mecánica invasiva son factores que indican una mayor propensión a presentar síntomas de la CPC^(1,2)^.

Se sugiere que los síntomas a largo plazo tras la recuperación de la fase aguda de la COVID-19 están asociados a la capacidad del virus para desencadenar una respuesta inflamatoria masiva. Con frecuencia, los síntomas de la CPC también están relacionados con la gravedad en la fase aguda de la enfermedad, pero también pueden presentarse tras una manifestación leve de la COVID-19, así como afectar a pacientes de todas las edades^(8)^. Además, los síntomas de la CPC pueden incluso agravar los síntomas preexistentes de las comorbilidades que los individuos presentaban previamente^(1)^.

Debido a las características de los síntomas de la CPC, que son comunes a otras enfermedades, el diagnóstico de esta afección suele pasarse por alto y resulta complejo. Actualmente, el mayor desafío en la atención a pacientes con CPC es el hecho de que esta enfermedad no puede considerarse una entidad clínica única, por lo que requiere un tratamiento multidisciplinario integrado, debido a la diversidad de síntomas^(2)^. Por lo tanto, comprender el curso de esta enfermedad es fundamental para la recuperación y la mejora de la calidad de vida de las personas.

Además, es necesario comprender plenamente la etiología de la CPC ante la diversidad de síntomas y fenotipos. Hasta el momento, la literatura indica que la CPC se asocia principalmente con el sexo femenino, la edad avanzada, la vulnerabilidad socioeconómica, el estado de vacunación contra la COVID-19 y afecciones clínicas preexistentes, como la diabetes, la hipertensión y la obesidad^(2,3,8,9)^. Sin embargo, aún son escasos los estudios que comparan la manifestación de los síntomas del CPC entre personas con y sin comorbilidades crónicas, lo que dificulta la comprensión de la influencia de estas afecciones en la persistencia de los síntomas. Ante lo expuesto, este estudio tiene como objetivo analizar y comparar los síntomas del condición pos-COVID en pacientes con y sin comorbilidades crónicas, como diabetes, hipertensión y obesidad.

## MÉTODO

### Diseño del Estudio

Se trata de un estudio transversal y analítico, elaborado de acuerdo con las recomendaciones de *Strengthening The Reporting of Observational Studies in Epidemiology* (STROBE).

### Lugar

Este estudio se llevó a cabo en el municipio de São Carlos, ubicado en el estado de São Paulo. Los datos se obtuvieron de los servicios de Vigilancia en Salud y del Departamento de Gestión y Atención Ambulatoria, sector a cargo de los servicios de Atención Primaria de Salud (APS).

### Población y Criterios de Selección

Se realizó un cálculo de muestreo considerando todos los casos de COVID-19 diagnosticados en la población mayor de 18 años del municipio, correspondientes al período comprendido entre el 26/02/2020 y el 26/02/2024, mediante prueba rápida (PR) o reacción en cadena de la polimerasa con transcripción inversa (*Reverse Transcription Polymerase Chain Reaction* - RT-PCR). En la ciudad de São Carlos (SP), se notificaron 62 977 casos positivos de COVID-19 en personas mayores de 18 años durante el período de análisis de este estudio. La elección de la prevalencia se basó en datos de la literatura^(10)^, que indican que alrededor del 50 % de los casos de COVID-19 presentaron síntomas de la CPC. Se incluyeron en el estudio pacientes adultos mayores de 18 años que dieron positivo en la prueba de COVID-19 y regresaron a los servicios de la APS con síntomas de CPC, según la definición de la OMS, que describe los síntomas como recurrentes o fluctuantes durante un período mínimo de dos meses después de la infección por la enfermedad aguda^(4)^. Se excluyeron del estudio los pacientes cuyos expedientes médicos no estaban disponibles, que no residían en localidades adscritas a la APS o que habían fallecido. Las variables del estudio fueron sexo, edad, raza/color, comorbilidades, esquema de vacunación y los síntomas reportados.

### Cálculo del Tamaño de la Muestra

Para el cálculo de la muestra, se asumió un nivel de significancia del 5 % (α = 0,05), un error absoluto tolerable de 5 puntos porcentuales (d = 0,05) y una población finita estimada en N = 31 489 pacientes con CPC mayores de 18 años en el municipio de interés. Se utilizó una prevalencia esperada de p = 0,356, correspondiente a la frecuencia del síntoma de fatiga, una de las manifestaciones más reportadas en estudios previos sobre el CPC. Por lo tanto, el tamaño de la muestra adoptado en este estudio fue de n = 350. El cálculo se realizó mediante la siguiente ecuación:


$$n=\frac{z_{\frac{\left(1-\alpha \right)}{2}}^{2}Np(1-p)}{d^{2}(N-1)+z_{\frac{\left(1-\alpha \right)}{2}}^{2}p(1-p)}$$

Donde p es la prevalencia esperada, d es el error absoluto tolerable, N es el tamaño de la población y z es el valor de la distribución normal estándar correspondiente al nivel de confianza deseado.

### Recolección de Datos

Para la recolección de datos, se crearon hojas de cálculo en Microsoft Excel que contenían las variables de interés. Inicialmente, se crearon diferentes pestañas para recopilar los datos provenientes de las distintas bases de datos utilizadas en esta investigación, obtenidos del SisCOVID, el sistema de notificación de COVID-19 del municipio de São Carlos, en el estado de SP. En la siguiente pestaña, se insertaron los datos relativos al estado de vacunación, obtenidos de VaciVida, un sistema desarrollado por el estado de São Paulo. Por último, en la última pestaña, se insertaron los datos relativos a los síntomas de la COVID-19 obtenidos del Expediente Electrónico del Ciudadano (llamado PEC). Se adoptaron medidas para minimizar los sesgos, incluyendo la capacitación de los recopiladores de datos (dos estudiantes de licenciatura, dos de maestría y uno de doctorado en Ciencias de la Salud), el uso de criterios de inclusión y exclusión previamente definidos y la estandarización de la recolección de datos mediante una hoja de cálculo estructurada y digitalizada. Además, cabe destacar la elección del nivel de significación adecuado (α = 0,05), el ajuste para comparaciones múltiples, la aplicación de métodos estadísticos apropiados y un tamaño de muestra robusto.

Así, en primer lugar, se identificaron los casos confirmados de COVID-19 en el SisCOVID; posteriormente, se consultó en el PEC si el paciente infectado por el SARS-CoV-2 había regresado a la atención primaria de salud (APS) con informe de síntomas de la CPC, de acuerdo con la definición de la OMS^(4)^. A través del SisCOVID, también fue posible obtener información sobre consultas de usuarios con quejas de síntomas respiratorios después de la fase aguda de la enfermedad; por su parte, en el PEC se verificaron los síntomas de la CPC referidos por los usuarios, que afectaban a los diferentes sistemas orgánicos del cuerpo. Además, los datos relativos a la vacunación se obtuvieron en la página de VaciVida, junto con la vigilancia epidemiológica municipal.

### Análisis y Procesamiento de los Datos

Inicialmente, los datos se describieron mediante frecuencias absolutas y porcentuales (variables cualitativas) y mediante medidas de centralidad y dispersión, como la media, la desviación estándar, el mínimo, la mediana y el máximo (variables cuantitativas). Para estimar la razón de prevalencia bruta de los síntomas de la CPC en relación con las variables de interés, se utilizó el modelo de regresión de Poisson con varianza robusta^(11)^. En los análisis en los que la frecuencia de los síntomas era nula, se realizó la prueba exacta de Fisher. El uso de *Datos faltantes* se indicó en el pie de página de las tablas para garantizar la transparencia de los datos presentados, manteniendo el número de casos ausentes tal como se observaron, sin imputación. Todos los análisis se realizaron con la ayuda del software SAS 9.4^(12)^, adoptando un nivel de significación del 5 %.

### Aspectos Éticos

De conformidad con la Resolución n.º 510/2016, el proyecto fue aprobado por el Comité de Ética en Investigación de la Universidad Federal de São Carlos (CAAE: 67128023.4.0000.5504; CAAE: 74911623.5.0000.5504).

## RESULTADOS

Para obtener la muestra de 350 casos de condición pos-COVID, se analizaron 3.778 casos notificados y confirmados de COVID-19, lo que corresponde al 9,26 % de los casos de la enfermedad aguda en la población adulta del municipio de São Carlos, en el estado de SP. Los resultados muestran que el 67,1 % (235) eran mujeres, el 61,6 % (215) de raza blanca, el 48,2 % (169) presentaban comorbilidades, de los cuales el 30,3 % (106) tenían hipertensión arterial sistémica (HAS), el 15,1 % (53) padecían diabetes mellitus (DM) y el 16 % (56) tenían obesidad, con una edad promedio de 46,9 años (Tabla 1).

**Tabla 1:** Características demográficas y clínicas de la muestra del estudio – São Carlos, SP, Brasil, 2024.

Características	Frecuencia(n = 350)	Porcentaje(%)
**Sexo**		
Femenino	235	67,1
Masculino	115	32,9
**Edad**		
Media	46,9 [15,8]	–
Mediana (mínimo y máximo)	47,0 [14,0 y 88,0]	–
**Grupo de edad**		
< 60 años	270	77,1 %
≥ 60 años	80	22,9 %
**Raza/Color**		
Amarilla	1	0,3
Blanco	215	61,6
Marrón	105	30,1
Negro	28	8,0
Falta	1	–
**Comorbilidades**		
Hemoglobinopatías	1	0,3
Cirrosis hepática	0	0
Enfermedades cardiovasculares	17	4,9
Enfermedades neurológicas	4	1,1
Diabetes mellitus (tipo 1 y tipo 2)	53	15,1
Enfermedad renal crónica	4	1,1
Hipertensión arterial sistémica	106	30,3
Inmunosuprimidos	0	0
Obesidad	56	16
Enfermedades pulmonares crónicas graves	21	6,0
Otros	51	14,6

La prevalencia de los síntomas de la CPC, teniendo en cuenta la ausencia o la presencia de comorbilidades, se redujo en un 58 % en el caso de la secreción nasal (IC del 95 %: 0,23;0,78), en un 31 % para el dolor de cabeza (IC del 95 %: 0,49; 0,98) y en un 46 % para el dolor de garganta (IC del 95 %: 0,34; 0,85) en personas sin comorbilidades (Tabla 2).

**Tabla 2:** Comparación de la presencia de síntomas de la condición pos-COVID, teniendo en cuenta la presencia de comorbilidades en la muestra del estudio – São Carlos, SP, Brasil, 2024.

Descripción de los síntomas del síndrome pos-COVID	Sin comorbilidades (n = 181)	Con comorbilidades (n = 169)	Razón de prevalencia [IC del 95 %]*	Valor p^†^
Alteración del ciclo menstrual^‡^	6 (3,6 %)	2 (1,2 %)	0,34 (0,07; 1,68)	0,19
Ansiedad^‡^	11 (6,6 %)	10 (6,2 %)	0,94 (0,41; 2,15)	0,88
Cansancio	32 (17,1 %)	25 (14,8 %)	0,84 (0,52; 1,35)	0,47
Secreción nasal	33 (18,2 %)	13 (7,7 %)	0,42 (0,23; 0,78)	<0,01
Diarrea	1 (0,6 %)	3 (1,8 %)	3,21 (0,34; 30,59)	0,31
Dificultad para concentrarse^‡^	3 (1,8 %)	2 (1,2 %)	0,69 (0,12; 4,06)	0,68
Dolor abdominal^‡^	12 (7,2 %)	9 (5,6 %)	0,77 (0,33; 1,79)	0,55
Dolor articular^‡^	4 (2,4 %)	3 (1,9 %)	0,77 (0,18; 3,4)	0,73
Dolor de cabeza	59 (32,6 %)	38 (22,5 %)	0,69 (0,49; 0,98)	0,04
Dolor de garganta	44 (24,3 %)	22 (13,0 %)	0,54 (0,34; 0,85)	<0,01
Dolor en las extremidades inferiores^‡^	14 (8,4 %)	25 (15,4 %)	1,84 (0,99; 3,41)	0,05
Dolor en las extremidades superiores^‡^	6 (3,6 %)	9 (5,6 %)	1,55 (0,56; 4,25)	0,40
Dolor muscular	28 (15,5 %)	23 (13,6 %)	0,88 (0,53; 1,47)	0,62
Dolor de espalda/lumbar^‡^	19 (11,4 %)	11 (6,8 %)	0,6 (0,29; 1,21)	0,15
Dolor de oído^‡^	0 (0 %)	1 (0,6 %)	–	0,49^§^
Dolor en el pecho^‡^	8 (4,8 %)	9 (5,6 %)	1,16 (0,46; 2,93)	0,75
Dolor en el tórax^‡^	8 (4,8 %)	5 (3,1 %)	0,64 (0,22; 1,93)	0,43
Dificultad para respirar	22 (12,2 %)	34 (20,1 %)	1,66 (1,01; 2,71)	0,05
Hormigueo/palpitaciones en las extremidades inferiores^‡^	7 (4,2 %)	9 (5,6 %)	1,33 (0,51; 3,47)	0,57
Hormigueo/pulsaciones en las extremidades superiores^‡^	6 (3,6 %)	9 (5,6 %)	1,55 (0,56; 4,25)	0,40
Debilidad o pérdida de fuerza muscular^‡^	12 (7,2 %)	18 (11,1 %)	1,55 (0,77; 3,11)	0,22
Insomnio^‡^	9 (5,4 %)	10 (6,2 %)	1,15 (0,48; 2,75)	0,76
Fiebre	19 (10,5 %)	15 (8,9 %)	0,85 (0,44; 1,61)	0,61
Náuseas	6 (3,3 %)	2 (1,2 %)	0,36 (0,07; 1,74)	0,20
Odinofagia^‡^	2 (1,2 %)	0 (0 %)	–	0,50^§^
Pérdida de memoria/confusión^‡^	5 (3,0 %)	10 (6,2 %)	2,06 (0,72; 5,9)	0,18
Pérdida del olfato	5 (2,8 %)	2 (1,2 %)	0,43 (0,08; 2,18)	0,31
Pérdida del gusto	3 (1,7 %)	1 (0,6 %)	0,36 (0,04; 3,4)	0,37
Caída del cabello^‡^	19 (11,4 %)	11 (6,8 %)	0,6 (0,29; 1,21)	0,15
Queja odontológica por pérdida dental tras la COVID-19 ^‡^	2 (1,2 %)	1 (0,6 %)	0,52 (0,05; 5,63)	0,59
Tristeza/depresión^‡^	5 (3,0 %)	10 (6,2 %)	2,06 (0,72; 5,9)	0,18
Mareos^‡^	7 (4,2 %)	8 (4,9 %)	1,18 (0,44; 3,17)	0,75
Tos	44 (24,3 %)	42 (24,9 %)	1,02 (0,71; 1,48)	0,91
Vómitos	9 (5,0 %)	3 (1,8 %)	0,36 (0,1; 1,3)	0,12

*IC = Intervalo de confianza;

^†^Valor p = Nivel de significación;

^‡^Datos faltantes: 20;

^§^Valor correspondiente a la prueba exacta de Fisher.

Entre las comorbilidades más frecuentes en la población del estudio, se analizó la relación entre la presencia de síntomas de la CPC y la HTA, la DM y la obesidad, prestando especial atención a la primera. Así, en relación con la DM, cabe destacar que la ansiedad solo se mencionó en la muestra que no presentaba esta comorbilidad. Por su parte, la prevalencia de la manifestación de los síntomas del CPC se redujo en un 50 % en el caso del dolor de cabeza (IC del 95 %: 0,26; 0,98) y en un 64 % en el caso del dolor de garganta (IC del 95 %: 0,14; 0,95) en personas no diabéticas (Tabla 3).

**Tabla 3:** Comparación de la presencia de síntomas de la condición pos COVID teniendo en cuenta el diagnóstico de DM de la población del estudio – São Carlos, SP, Brasil, 2024.

Descripción de los síntomas del síndrome pos-COVID	No diabéticos [A] (n = 297)	Diabéticos [B] (n = 53)	Razón de prevalencia [B/A] [IC del 95 %]*	Valor p^†^
Alteración del ciclo menstrual^‡^	8 (2,9 %)	0 (0 %)	–	0,36^§^
Ansiedad^‡^	21 (7,6 %)	0 (0 %)	–	0,03^§^
Cansancio	49 (16,5 %)	8 (15,1 %)	0,91 (0,46; 1,82)	0,80
Secreción nasal	44 (14,9 %)	2 (3,8 %)	0,25 (0,06; 1,02)	0,05
Diarrea	2 (0,7 %)	2 (3,8 %)	5,6 (0,81; 38,92)	0,08
Dificultad para concentrarse^‡^	5 (1,8 %)	0 (0 %)	–	0,99^§^
Dolor abdominal^‡^	17 (6,2 %)	4 (7,5 %)	1,23 (0,43; 3,5)	0,70
Dolor articular^‡^	5 (1,8 %)	2 (3,8 %)	2,08 (0,42; 10,46)	0,37
Dolor de cabeza	89 (30,0 %)	8 (15,1 %)	0,5 (0,26; 0,98)	0,04
Dolor de garganta	62 (20,9 %)	4 (7,5 %)	0,36 (0,14; 0,95)	0,04
Dolor en las extremidades inferiores^‡^	33 (12,0 %)	6 (11,3 %)	0,95 (0,42; 2,15)	0,90
Dolor en las extremidades superiores^‡^	10 (3,6 %)	5 (9,4 %)	2,6 (0,93; 7,31)	0,07
Dolor muscular	46 (15,5 %)	5 (9,4 %)	0,61 (0,25; 1,46)	0,27
Dolor de espalda/lumbar^‡^	25 (9,1 %)	5 (9,4 %)	1,04 (0,42; 2,6)	0,93
Dolor de oído^‡^	0 (0 %)	1 (1,9 %)	–	0,16^§^
Dolor en el pecho^‡^	15 (5,4 %)	2 (3,8 %)	0,69 (0,16; 2,95)	0,62
Dolor en el tórax^‡^	12 (4,3 %)	1 (1,9 %)	0,43 (0,06; 3,27)	0,42
Dificultad para respirar	44 (14,8 %)	12 (22,6 %)	1,53 (0,87; 2,7)	0,14
Hormigueo/palpitaciones en las extremidades inferiores^‡^	11 (4,0 %)	5 (9,4 %)	2,37 (0,86; 6,53)	0,10
Hormigueo/pulsaciones en las extremidades superiores^‡^	7 (2,5 %)	4 (7,5 %)	2,98 (0,9; 9,81)	0,07
Debilidad o pérdida de fuerza muscular^‡^	22 (8,0 %)	8 (15,1 %)	1,89 (0,89; 4,02)	0,10
Insomnio^‡^	17 (6,2 %)	2 (3,8 %)	0,61 (0,15; 2,57)	0,50
Fiebre	32 (10,8 %)	2 (3,8 %)	0,35 (0,09; 1,42)	0,14
Náuseas	8 (2,7 %)	0 (0 %)	–	0,61^§^
Odinofagia^‡^	2 (0,7 %)	0 (0 %)	–	0,99^§^
Pérdida de memoria/confusión^‡^	12 (4,3 %)	3 (5,7 %)	1,3 (0,38; 4,46)	0,67
Pérdida del olfato	7 (2,4 %)	0 (0 %)	–	0,60^§^
Pérdida del gusto	4 (1,3 %)	0 (0 %)	–	0,99^§^
Caída del cabello^‡^	25 (9,1 %)	5 (9,4 %)	1,04 (0,42; 2,6)	0,93
Queja odontológica por pérdida dental tras la COVID-19^‡^	3 (1,1 %)	0 (0 %)	–	0,99^§^
Tristeza/depresión^‡^	13 (4,7 %)	2 (3,8 %)	0,8 (0,19; 3,45)	0,77
Mareos^‡^	12 (4,3 %)	3 (5,7 %)	1,3 (0,38; 4,46)	0,67
Tos	75 (25,3 %)	11 (20,8 %)	0,82 (0,47; 1,44)	0,49
Vómitos	11 (3,7 %)	1 (1,9 %)	0,51 (0,07; 3,86)	0,51

*IC = Intervalo de confianza;

^†^Valor p = Nivel de significación;

^‡^Datos faltantes: 20;

^§^Valor correspondiente a la prueba exacta de Fisher.

Los resultados muestran que las personas hipertensas presentaron una prevalencia dos veces mayor de manifestar debilidad y/o pérdida de fuerza muscular en comparación con las personas normotensas (IC del 95 %: 1,13; 4,38); además, las personas hipertensas no referían la presencia de náuseas, odinofagia ni vómitos (Tabla 4).

**Tabla 4:** Comparación de la presencia de síntomas de condición pos COVID teniendo en cuenta el diagnóstico de HTA en la población del estudio – São Carlos, SP, Brasil, 2024.

Descripción de los síntomas del síndrome pos-COVID	Personas con presión arterial normal [A] (n = 244)	Hipertensos [B] (n = 106)	Razón de prevalencia [B/A] [IC del 95 %]*	Valor p^†^
Alteración del ciclo menstrual^‡^	7 (3,1 %)	1 (1,0 %)	0,32 (0,04; 2,55)	0,28
Ansiedad^‡^	15 (6,6 %)	6 (5,9 %)	0,89 (0,36; 2,23)	0,80
Cansancio	40 (16,4 %)	17 (16,0 %)	0,98 (0,58; 1,64)	0,93
Secreción nasal	34 (14,0 %)	12 (11,3 %)	0,81 (0,44; 1,5)	0,50
Diarrea	3 (1,2 %)	1 (0,9 %)	0,77 (0,08; 7,29)	0,82
Dificultad para concentrarse^‡^	4 (1,8 %)	1 (1,0 %)	0,56 (0,06; 4,92)	0,60
Dolor abdominal^‡^	13 (5,7 %)	8 (7,8 %)	1,37 (0,59; 3,2)	0,47
Dolor articular^‡^	5 (2,2 %)	2 (2,0 %)	0,89 (0,18; 4,51)	0,89
Dolor de cabeza	71 (29,1 %)	26 (24,5 %)	0,84 (0,57; 1,24)	0,39
Dolor de garganta	51 (20,9 %)	15 (14,2 %)	0,68 (0,1; 1,15)	0,15
Dolor en las extremidades inferiores^‡^	22 (9,7 %)	17 (16,7 %)	1,72 (0,96; 3,1)	0,07
Dolor en las extremidades superiores^‡^	10 (4,4 %)	5 (4,9 %)	1,11 (0,39; 3,17)	0,84
Dolor muscular	36 (14,8 %)	15 (14,2 %)	0,96 (0,55; 1,67)	0,88
Dolor de espalda/lumbar^‡^	23 (10,1 %)	7 (6,9 %)	0,68 (0,3; 1,53)	0,35
Dolor de oído^‡^	0 (0 %)	1 (1,0 %)	–	0,31^§^
Dolor en el pecho^‡^	13 (5,7 %)	4 (3,9 %)	0,68 (0,23; 2,05)	0,50
Dolor en el tórax^‡^	12 (5,3 %)	1 (1,0 %)	0,19 (0,02; 1,41)	0,10
Dificultad para respirar	35 (14,3 %)	21 (19,8 %)	1,38 (0,85; 2,26)	0,20
Hormigueo/palpitaciones en las extremidades inferiores^‡^	11 (4,8 %)	5 (4,9 %)	1,01 (0,36; 2,84)	0,98
Hormigueo/pulsaciones en las extremidades superiores^‡^	8 (3,5 %)	3 (2,9 %)	0,83 (0,23; 3,08)	0,79
Debilidad o pérdida de fuerza muscular^‡^	15 (6,6 %)	15 (14,7 %)	2,23 (1,13; 4,38)	0,02
Insomnio^‡^	11 (4,8 %)	8 (7,8 %)	1,62 (0,67; 3,9)	0,28
Fiebre	21 (8,6 %)	13 (12,3 %)	1,43 (0,74; 2,74)	0,29
Náuseas	8 (3,3 %)	0 (0 %)	–	0,11^§^
Odinofagia^‡^	2 (0,9 %)	0 (0 %)	–	0,99^§^
Pérdida de memoria/confusión^‡^	8 (3,5 %)	7 (6,9 %)	1,95 (0,73; 5,23)	0,19
Pérdida del olfato	5 (2,0 %)	2 (1,9 %)	0,92 (0,18; 4,67)	0,92
Pérdida del gusto	3 (1,2 %)	1 (0,9 %)	0,77 (0,08; 7,29)	0,82
Caída del cabello^‡^	25 (11,0 %)	5 (4,9 %)	0,45 (0,18; 1,13)	0,09
Queja odontológica por pérdida dental tras la COVID-19^‡^	2 (0,9 %)	1 (1,0 %)	1,11 (0,1; 12,13)	0,93
Tristeza/depresión^‡^	8 (3,5 %)	7 (6,9 %)	1,95 (0,73; 5,23)	0,19
Mareos^‡^	10 (4,4 %)	5 (4,9 %)	0,45 (0,18; 1,13)	0,09
Tos	55 (22,5 %)	31 (29,2 %)	1,3 (0,89; 1,89)	0,18
Vómitos	12 (4,9 %)	0 (0 %)	–	0,02^§^

*IC = Intervalo de confianza;

^†^Valor p = Nivel de significación;

^‡^Faltan 20;

^§^Valor correspondiente a la prueba exacta de Fisher.

Las personas con obesidad no informaron de la presencia de secreción nasal, dolor de oído, insomnio, pérdida del olfato y del gusto, molestias dentales ni vómitos. Además, la diarrea (IC del 95 %: 1,67; 148,7) y el hormigueo o palpitaciones en las extremidades superiores (IC del 95 %: 1,89; 18,9) fueron más frecuentes en las personas obesas. Por otra parte, la prevalencia de los síntomas de la condición pos COVID en personas obesas se redujo en un 59 % en el caso del dolor de cabeza (IC del 95 %: 0,2; 0,83) y en un 84 % en el caso del dolor de garganta (IC del 95 %: 0,04; 0,65) en comparación con las personas de peso normal (Tabla 5).

**Tabla 5:** Comparación de la presencia de síntomas del síndrome pos-COVID, teniendo en cuenta el diagnóstico de obesidad de la muestra del estudio – São Carlos, SP, Brasil, 2024.

Descripción de los síntomas del síndrome pos-COVID	Personas con peso normal [A] (n = 294)	Obesos [B] (n = 56)	Razón de prevalencia [B/A] [IC del 95 %]*	Valor p^†^
Alteración del ciclo menstrual^‡^	7 (2,6 %)	1 (1,8 %)	0,71 (0,09; 5,67)	0,75
Ansiedad^‡^	15 (5,5 %)	6 (109 %)	1,99 (0,81; 4,91)	0,13
Cansancio	51 (17,3)	6 (10,7 %)	0,62 (0,28; 1,37)	0,24
Secreción nasal	46 (15,7 %)	0 (0 %)	–	<0,01^§^
Diarrea	1 (0,3 %)	3 (5,4 %)	15,75 (1,67; 148,7)	0,02
Dificultad para concentrarse^‡^	4 (1,5 %)	1 (1,8 %)	1,25 (0,14; 0,93)	0,84
Dolor abdominal^‡^	20 (7,3 %)	1 (1,8 %)	0,25 (0,03; 1,82)	0,17
Dolor articular^‡^	6 (2,2 %)	1 (1,8 %)	0,83 (0,1; 6,76)	0,86
Dolor de cabeza	90 (30,6 %)	7 (1,5 %)	0,41 (0,2; 0,83)	0,01
Dolor de garganta	64 (21,8 %)	2 (3,6 %)	0,16 (0,04; 0,65)	0,01
Dolor en las extremidades inferiores^‡^	29 (10,6 %)	10 (18,2 %)	1,72 (0,89; 3,32)	0,11
Dolor en las extremidades superiores^‡^	11 (4,0 %)	4 (7,3 %)	1,81 (0,6; 5,48)	0,29
Dolor muscular	47 (16,0 %)	4 (7,1 %)	0,45 (0,17; 1,19)	0,11
Dolor de espalda/lumbar^‡^	26 (9,5 %)	4 (7,3 %)	0,77 (0,28; 2,11)	0,61
Dolor de oído^‡^	1 (0,4 %)	0 (0 %)	–	0,99^§^
Dolor en el pecho^‡^	16 (5,8 %)	1 (1,8 %)	0,31 (0,04; 2,3)	0,25
Dolor en el tórax^‡^	12 (4,4 %)	1 (1,8 %)	0,42 (0,06; 3,13)	0,39
Dificultad para respirar	51 (17,3 %)	5 (8,9 %)	0,51 (0,22; 1,23)	0,14
Hormigueo/pulsaciones en las extremidades inferiores^‡^	12 (4,4 %)	4 (7,3 %)	1,66 (0,56; 4,96)	0,36
Hormigueo/pulsaciones en las extremidades superiores^‡^	5 (1,8 %)	6 (10,9 %)	5,98 (1,89; 18,9)	<0,01
Debilidad o pérdida de fuerza muscular^‡^	25 (9,1 %)	5 (9,1 %)	1 (0,4; 2,49)	0,99
Insomnio^‡^	19 (6,9 %)	0 (0 %)	–	0,05^§^
Fiebre	33 (11,2 %)	1 (1,8 %)	0,16 (0,02; 1,14)	0,07
Náuseas	6 (2,0 %)	2 (3,6 %)	1,75 (0,36; 8,45)	0,49
Odinofagia^‡^	2 (0,7 %)	0 (0 %)	–	0,99^§^
Pérdida de memoria/confusión^‡^	11 (4,0 %)	4 (7,3 %)	1,81 (0,6; 5,48)	0,29
Pérdida del olfato	7 (2,4 %)	0 (0 %)	–	0,60^§^
Pérdida del gusto	4 (1,4 %)	0 (0 %)	–	0,99^§^
Caída del cabello^‡^	25 (9,1 %)	5 (9,1 %)	1 (0,4; 2,49)	0,99
Queja odontológica por pérdida dental tras la COVID-19^‡^	3 (1,1 %)	0 (0 %)	–	0,99^§^
Tristeza/depresión^‡^	12 (4,4 %)	3 (5,5 %)	1,25 (0,36; 4,27)	0,73
Mareos^‡^	13 (4,7 %)	2 (3,6 %)	0,77 (0,18; 3,3)	0,72
Tos	78 (26,5 %)	8 (14,3 %)	0,54 (0,28; 1,05)	0,07
Vómitos	12 (4,1 %)	0 (0 %)	–	0,23^§^

*IC = Intervalo de confianza;

^†^Valor p = Nivel de significación;

^‡^Faltan 20;

^§^Valor correspondiente a la prueba exacta de Fisher.

Este análisis puso de manifiesto que las personas sin DM presentaron una menor prevalencia en la manifestación de síntomas como dolor de cabeza y dolor de garganta, mientras que las personas con hipertensión mostraron una mayor propensión a la debilidad muscular. Por su parte, las personas obesas presentaron una mayor prevalencia en el desarrollo de diarrea y hormigueo en las extremidades superiores, pero una menor prevalencia de dolor de cabeza y de garganta. Estos resultados resaltan la influencia de las comorbilidades en los síntomas de la CPC, lo que sugiere que la presencia de DM, HAS y obesidad puede modificar el perfil sintomático según cada afección. Al igual que los resultados de este estudio, la literatura también destaca que las comorbilidades más frecuentes en personas con CPC son la DM, la HAS y la obesidad^(13)^.

En este contexto, con el fin de aclarar las relaciones establecidas entre la presencia de comorbilidades, como la DM, la HAS y la obesidad, y la aparición de los síntomas de la CPC, se sugiere una relación con el proceso inflamatorio crónico posterior a la infección por el SARS-CoV-2. Además de la mayor prevalencia de personas con DM que presentan un empeoramiento de la COVID-19, este grupo se ve afectado con mayor frecuencia por la CPC, siendo más susceptible a las consecuencias a largo plazo que las personas sin DM. Se sabe que los pacientes con DM presentan una inflamación crónica debido a una señalización de la insulina alterada, lo que provoca una disminución de las citocinas antiinflamatorias y una mayor expresión de las citocinas proinflamatorias, como el factor de necrosis tumoral alfa (TNF-α), la interleucina 6 (IL-6) y la interleucina beta (IL-1β). Así, la actividad de las citocinas inhibe la señalización de la insulina; en consecuencia, se produce un aumento de la resistencia a la insulina, lo que favorece la insuficiencia respiratoria y el colapso cardiopulmonar^(3)^.

Es notorio que la pandemia de COVID-19 ha influido en el control de la DM, así como en la progresión de la prediabetes y el aumento del número de casos de la enfermedad debido al uso de corticosteroides, ya que la terapia con altas dosis ha provocado hiperglucemia grave^(1,14,15)^. Los agonistas de medicamentos utilizados para el control glicémico, como la pioglitazona, aumentan la secreción del gen ECA 2; hay indicios de que este medicamento inhibe la secreción de citocinas proinflamatorias y aumenta la de citocinas antiinflamatorias, además de atenuar la lesión pulmonar y reducir la reacción fibrótica pulmonar. Además, las personas con diabetes que toman metformina tienen una menor probabilidad de contraer infecciones por COVID-19^(3,16,17)^ y, en consecuencia, esto puede influir en que no desarrollen los síntomas de la CPC.

Un estudio realizado en el Reino Unido mostró que las personas con DM (tipo 1 o 2) se asociaron con una mayor probabilidad de un diagnóstico registrado de CPC. Al analizar la prevalencia de la CPC entre hombres con DM2 y sus controles emparejados, la prevalencia fue menor en los controles emparejados: 0,46 % frente a 0,54 % en comparación con los hombres sin DM2 (p = 0,008).

Además, el estudio mencionado indicó que la prevalencia de la CPC fue de 0,54 % en hombres con DM2 y de 0,53 % en mujeres con DM2, sin diferencias estadísticamente significativas entre los géneros. Una hipótesis para explicar la similitud en la prevalencia entre hombres y mujeres con DM y síntomas de la CPC podría estar relacionada con el seguimiento regular de su afección crónica, lo que los hace potencialmente más propensos a reportar síntomas, incluidos los relacionados con la CPC, aumentando así la probabilidad de detección de esta afección^(18)^.

En este estudio, las personas sin DM presentaron prevalencias más bajas en cuanto al desarrollo de dolor de cabeza y dolor de garganta como síntomas de la CPC. La fisiopatología del dolor en la CPC es multifactorial e involucra varios mecanismos. El dolor persistente en pacientes con CPC puede explicarse por factores como la inflamación sistémica y en el sistema nervioso central, que sensibilizan los receptores del dolor, además de alteraciones en la perfusión cerebral y daños neuronales directos causados por el virus. Las alteraciones en los neurotransmisores, como la serotonina y la dopamina, y los factores psicológicos, como el estrés y la ansiedad, también agravan el dolor. Estos mecanismos, ya sea de forma aislada o combinada, contribuyen a los dolores persistentes^(19)^.

En Estados Unidos, un análisis realizado en personas con DM que habían tenido COVID-19 indicó que el control glicémico inadecuado y los niveles elevados de hemoglobina glicosilada (HbA1c ≥ 8 %) aumentan el riesgo de aparición de nuevos síntomas recurrentes de la CPC, especialmente síntomas respiratorios y confusión mental, entre 30 y 180 días después de la infección por COVID-19, lo que sugiere secuelas persistentes asociadas a infecciones agudas. Sin embargo, los mecanismos que explican estos hallazgos son hipotéticos y apuntan a la reducción del estrés oxidativo y la inflamación; por lo tanto, el tratamiento de la enfermedad crónica contribuiría a reducir el riesgo de desarrollar la CPC^(20)^.

En cuanto al análisis de la hipertensión y la CPC, en este estudio, las personas hipertensas presentaron una mayor prevalencia de desarrollar síntomas de debilidad y/o pérdida de fuerza muscular en comparación con las personas normotensas. Un estudio realizado en España mostró que la hipertensión no se asoció con ningún síntoma específico de la CPC; sin embargo, una mayor proporción de pacientes hipertensos reportó ≥ 3 síntomas de la CPC en comparación con los pacientes normotensos^(21)^. Las manifestaciones musculoesqueléticas asociadas a la CPC incluyen mialgia, dolor en las articulaciones y debilidad muscular. Los pacientes que utilizaron corticosteroides durante la fase aguda de la enfermedad y las personas hospitalizadas en UTI pueden desarrollar debilidad muscular hasta un año después de la infección. También se observó debilidad muscular en personas con un cuadro inicial leve de COVID-19, aunque no se ha aclarado si esto es resultado únicamente del desacondicionamiento o del desuso muscular^(22)^.

Los mecanismos subyacentes a la debilidad muscular aún no se comprenden por completo, pero algunas explicaciones posibles incluyen daños directos a las neuronas motoras y a los músculos adyacentes causados por el virus o por la respuesta inmunológica durante la infección aguda por COVID-19. Además, el virus puede dañar las mitocondrias e interferir en la cadena de transporte de electrones, que es responsable de la producción de energía en los músculos. Este proceso contribuye a la fatiga, la debilidad y la reducción de la tolerancia al ejercicio^(23)^.

En el contexto de la hospitalización por COVID-19, la estancia prolongada en la UTI se ha asociado con una pérdida sustancial de masa muscular, con una reducción promedio del 18,5 % en el volumen del músculo recto femoral hasta el séptimo día de hospitalización. También se señala que la sedación profunda, esencial para la implementación de la ventilación mecánica invasiva (VMI), compromete la función cardiopulmonar e induce un estado de inmovilidad total, lo que afecta negativamente a la musculatura esquelética. Cuando la sedación por sí sola no es suficiente para garantizar la VMI, se utilizan bloqueadores neuromusculares para sincronizar al paciente con el ventilador; sin embargo, estos agentes interrumpen la transmisión de impulsos en las células musculares. La persistencia del bloqueo neuromuscular puede provocar parálisis prolongada, miopatía y debilidad muscular generalizada^(24)^. De este modo, las secuelas musculares observadas en la CPC se derivan de una disfunción muscular persistente que se inicia en la fase aguda de la enfermedad, principalmente debido a la disminución de la síntesis proteica muscular, agravada por un estado hiperinflamatorio, hipoxemia, infección de las células musculares y desregulación del sistema renina-angiotensina (SRA).

En cuanto a las personas obesas, en este estudio los obesos presentaron una mayor prevalencia de desarrollar diarrea y hormigueo y/o palpitaciones en las extremidades superiores. Se constató que los pacientes con un Índice de Masa Corporal (IMC) basal en el rango de sobrepeso u obesidad tenían un mayor riesgo de presentar síntomas de la CPC, y aquellos con un IMC superior a 30 kg/m^2^ presentaban un riesgo un 10 % mayor de reportar síntomas de la CPC en comparación con los pacientes que tenían un IMC comprendido entre 18,5 y 25 kg/m^2(25)^.

Asimismo, la obesidad se caracteriza por el desarrollo de un estado inflamatorio crónico, considerado un componente clave en la resistencia a la insulina. Así, la expansión del tejido adiposo se produce como consecuencia del exceso calórico y del aumento en la infiltración de células inmunitarias, lo que promueve una respuesta proinflamatoria posterior mediante el aumento de TNF-α, IL-1β o IL-6. Además, la activación del receptor Toll-Like (TLR) debido a la infección por patógenos, como el SARS-CoV-2, induce inflamación, que se manifiesta en el músculo esquelético y en el tejido adiposo^(26)^.

Dado que el SARS-CoV-2 es un virus envuelto, es decir, está rodeado por una doble capa lipídica, desempeña un papel importante en el ciclo de vida viral a través de su metabolismo lipídico. Así, el virus utiliza la envoltura lipídica para facilitar la invasión del huésped, con el objetivo de promover la síntesis lipídica y la modificación de la señalización de las células del huésped, lo que culmina en una “tormenta de citoquinas” debido a la activación excesiva de las células inmunitarias que desregula la producción de lípidos. De este modo, las lipoproteínas de alta densidad (HDL), que se encuentran en cantidades reducidas, no pueden unirse ni neutralizar los lípidos asociados a los patógenos que provocan inflamación crónica, como ocurre en la CPC^(15)^. Por lo tanto, la asociación entre la obesidad y la CPC provoca alteraciones multisistémicas, lo que fomenta una inflamación sistémica que afecta a los órganos y a los tejidos musculares^(27)^. Síntomas como la diarrea se deben al daño en las barreras epiteliales intestinales causado por el SARS-CoV-2^(17)^ y al daño epitelial en los vasos sanguíneos^(28)^, lo que explica los síntomas de hormigueo en las extremidades superiores.

Cabe destacar que las personas con comorbilidades, como la diabetes mellitus (DM), la HAS y la obesidad, tienen una prevalencia significativamente mayor de ser hospitalizadas debido a la COVID-19. Las comorbilidades comprometen el sistema inmunológico y aumentan la vulnerabilidad a complicaciones graves, como insuficiencia respiratoria, síndrome de dificultad respiratoria aguda y problemas cardiovasculares. Estos factores hacen que la infección sea más difícil de controlar y aumentan la necesidad de hospitalización. Además, la presencia de comorbilidades puede prolongar la duración de la hospitalización y aumentar el riesgo de secuelas a largo plazo, como la CPC^(24)^.

Sin embargo, aunque la presencia de comorbilidades se considera un factor de riesgo para la CPC, la literatura ha señalado que la vacunación contra la COVID-19 es un factor protector frente al desarrollo de la enfermedad pos-COVID. Un análisis indicó que la vacunación antes de la infección viral con dos dosis se asoció con una reducción del 24 % en el riesgo de CPC, y que la vacunación después de la infección viral con una dosis se asoció con una reducción del 15 % en el riesgo de CPC^(29)^. Por lo tanto, fomentar la vacunación contra la COVID-19 en pacientes con CPC es crucial, ya que, además de que la vacunación disminuye el riesgo de complicaciones graves en la fase aguda de la enfermedad, puede mejorar la calidad de vida al reducir los síntomas persistentes y ayudar a disminuir la transmisión del virus. Por lo tanto, la vacuna desempeña un papel esencial en el manejo y la prevención de la CPC^(29,30)^.

Este estudio presenta limitaciones, como la subestimación de la prevalencia real de la CPC en la muestra, dada la calidad de los datos registrados en los expedientes médicos electrónicos; además, aunque los resultados encontrados sugieren asociaciones relevantes entre las variables analizadas, no es posible inferir causalidad en un diseño transversal, por lo que los hallazgos deben interpretarse con cautela en cuanto a su generalización. Sin embargo, los hallazgos de este estudio evidencian que las personas con HAS y obesidad pueden presentar una mayor prevalencia de síntomas, respectivamente, debilidad y/o pérdida de fuerza muscular, diarrea y hormigueo y/o palpitaciones en las extremidades superiores. De este modo, los resultados de este estudio contribuyen significativamente al avance científico al proporcionar una comprensión más detallada de cómo las comorbilidades específicas influyen en los síntomas y ofrecen información valiosa para el desarrollo de estrategias de tratamiento más específicas. Estos hallazgos pueden mejorar los enfoques clínicos, centrándose en intervenciones adaptadas a cada perfil de paciente, lo que mejora el manejo y la calidad de vida de las personas afectadas por la CPC.

## CONCLUSIÓN

Los resultados de este estudio muestran una mayor frecuencia de síntomas de la CPC entre las mujeres, de raza blanca, con una edad promedio de 46,9 años y con comorbilidades, siendo las más comunes la DM, la HAS y la obesidad. Asimismo, cabe destacar que las personas con DM presentaron una menor prevalencia de desarrollar dolor de cabeza y dolor de garganta en comparación con los no diabéticos; los hipertensos tienden a presentar debilidad y/o pérdida de fuerza muscular en comparación con los normotensos, y los obesos tuvieron mayor probabilidad de presentar diarrea y hormigueo y/o punzadas en las extremidades superiores. Estos resultados resaltan la importancia de las comorbilidades en el perfil sintomático de la CPC, lo que sugiere la necesidad de estrategias específicas para el manejo de estas personas. Se sugiere que los estudios futuros sobre la presentación de síntomas de la CPC en personas con comorbilidades incluyan variables relacionadas con la edad, el sexo, el origen étnico, la adherencia al tratamiento recomendado para la enfermedad preexistente y el estado del esquema de vacunación contra la COVID-19, factores que pueden influir en la prevalencia y la manifestación de los síntomas en la CPC.

## Data Availability

Todo el conjunto de datos que respalda los resultados de este estudio se publicó en el propio artículo.
